# The Prospective Application of Melatonin in Treating Epigenetic Dysfunctional Diseases

**DOI:** 10.3389/fphar.2022.867500

**Published:** 2022-05-20

**Authors:** Seth Mikaye Monayo, Xin Liu

**Affiliations:** Department of Pharmacology (The State-Province Key Laboratories of Biomedicine-Pharmaceutics of China, Key Laboratory of Cardiovascular Research, Ministry of Education), College of Pharmacy, Harbin Medical University, Harbin, China

**Keywords:** epigenetic modification, DNA methylation, histone modification, non-coding RNAs, melatonin

## Abstract

In the past, different human disorders were described by scientists from the perspective of either environmental factors or just by genetically related mechanisms. The rise in epigenetic studies and its modifications, i.e., heritable alterations in gene expression without changes in DNA sequences, have now been confirmed in diseases. Modifications namely, DNA methylation, posttranslational histone modifications, and non-coding RNAs have led to a better understanding of the coaction between epigenetic alterations and human pathologies. Melatonin is a widely-produced indoleamine regulator molecule that influences numerous biological functions within many cell types. Concerning its broad spectrum of actions, melatonin should be investigated much more for its contribution to the upstream and downstream mechanistic regulation of epigenetic modifications in diseases. It is, therefore, necessary to fill the existing gaps concerning corresponding processes associated with melatonin with the physiological abnormalities brought by epigenetic modifications. This review outlines the findings on melatonin’s action on epigenetic regulation in human diseases including neurodegenerative diseases, diabetes, cancer, and cardiovascular diseases. It summarizes the ability of melatonin to act on molecules such as proteins and RNAs which affect the development and progression of diseases.

## 1 Introduction

Epigenetics can be redefined as any biological process that changes the genetic expression and activity minus alteration of the DNA sequence resulting in cellular modifications (Dupont et al., 2009). There are a few types of epigenetic modifications that have been uncovered: histone modifications, DNA methylation, and “RNA epigenetics.” Of the three, DNA methylation has been the most extensively studied modification in epigenetics and has been found to influence genetic stability, mutations, and gene expressions (Bird, 1986; Bell et al., 2011; Lind and Spagopoulou, 2018). Located strategically at position 5 of carbon on the CpG sites (cytosine and guanine separated by a phosphate) makes it available to convert cytosine to 5-methylcytosine. Due to heavy methylation in some regions than others, during cell division, genetic material transfer results in aberrantly programmed transcription inherited by the daughter cells aided by DNA methyltransferases (Edwards et al., 2017). Such modulations have been confirmed in many diseases making DNA methylation a very important agent in the study of the etiology of diseases (Wang et al., 2018a).

Histone modification is a post-translational process involving gene expression alterations through the small protein ‘tails’ from the nucleosomes. The covalent addition of these histone proteins including methylation, phosphorylation, sumoylation, acetylation, and ubiquitylation, have all been shown to be responsible for the epigenetic modifications (Paz et al., 2011). Aberrations in histone modifications can lead to the development and progression of diseases through disruption of gene regulations which could be transmitted from one generation to another (Renani et al., 2019).

The lately discovered epigenetic modification is the “RNA epigenetics” which includes the non-coding RNAs (miRNA, siRNA, lncRNA, lincRNA, and eRNA) and RNA methylation (N^6^-adenosine, 5-methylcytosine, pseudouridine, and N^1^-methyladenosine). The microRNA (miRNA) has been a notorious contributor to diseases when it binds to specific sites associated with the translation of mRNA, inhibiting the process. For instance, when binding occurs at the 3’ untranslated regions of mRNA, the actions of mRNA are suppressed (Sarshad et al., 2018). Collectively, epigenetic modifications play a key role in the biological processes and pathogenesis of diseases. Since its emergence, numerous studies on human diseases have been conducted in an attempt to design epigenetic therapies for the prevention and management of diseases.

Melatonin is a widely spread indoleamine derivative (chemically identified as N-acetyl-5-methoxytryptamine) hormone largely synthesized by the pineal gland and can also be found in small quantities in organs such as the retina, lens, gut, bone marrow, skin, and other solid organs with multifunctional facets (Acuña-Castroviejo et al., 2014). Because of its extensive distribution in diverse cells and well penetrability through bio-barriers, it is been reported to induce pathophysiological signals. For instance, in neurodegenerative disorders, supplementation of melatonin has been widely reported to alleviate cognition impairment through different mechanisms, including the normalization of brain insulin signaling (Xu et al., 2019), the elevation of antioxidant properties, restoration of antioxidants like glutathione (Paul et al., 2018), reduction of the amyloid-β (Aβ) and anti-inflammatory action (Lee et al., 2018a).

Favorable evidence has premised an antagonistic relationship between melatonin and insulin secretions observed in diabetic patients. Studies have demonstrated that the action of beta cells of the pancreas is suppressed by melatonin and vice versa (Li et al., 2018). That is, these two hormones tend to take turns to subdue each other, since the levels of melatonin are high at night in response to decreased insulin levels, and melatonin is low during the day in response to high levels of insulin. The presence of melatonin receptors MT1 and MT2 on the pancreases (Zibolka et al., 2015) provides a biochemical basis implicating the action of melatonin on insulin-dependent diabetic patients. Studies have revealed that melatonin improved aberrant glucose metabolism and decreased insulin secretion in diabetes (Li et al., 2018).

In the study of cancer, melatonin has exhibited positive results in the prevention and improvement of this disease. The vast knowledge of the hallmarks of cancer has expedited the understanding and development of different mechanisms involved in the treatment of cancer by melatonin. Mechanisms range from, targeting DNA repair, increasing the cytotoxicity of chemotherapy drugs, scavenging free radicals, and inhibiting tumor growth and metastasis (Chen et al., 2018). Studies performed on cardiovascular diseases have shown innumerable genes linked with the beneficial effects of melatonin receptors activation, suggesting its potential therapeutic action as it confers profound protective effects on the heart. Differentially expressed genes obtained via a networking analysis based on transcriptomes revealed transcriptional factors highly sensitive to melatonin treatment (Li et al., 2019a).

Based on the vast effects of melatonin on a variety of diseases, it is worth pondering the deep reason for the diverse functions and the common impression in these conditions. Regulation of epigenetic modification by melatonin seems to be the reasonable answer to the question. Yet, the study of the interaction between melatonin and epigenetics in human pathology is still in its infancy. There is no coherent and consistent picture to summarize the relativity of epigenetic modifications by melatonin currently. Therefore, we intend to narrow the gap by unraveling potential markers on the connections between previous and current work on the basic mechanisms involving epigenetic changes as a result of effects imparted by melatonin (both endogenous and exogenous) in pursuit of treatment of pathologies across the clinical spectrum. The information collated herein serves to account for the prospective formulation of ideas on melatonin’s function in alleviating various diseases. Furthermore, as a quick response guide, we have summarized in Table 1, the genes involved in the pathway and the mechanism of action by which melatonin alleviates these epigenetic-modulated diseases.

**TABLE 1:** Summary of the effects of melatonin on epigenetic-modulated diseases.

Epigenetic-modulated disease	Genes in pathway	Melatonin’s mechanism of action	Reference
Alzheimer’s disease	MAPK, ERK1/2, p300, CREBBP	Increased p300 HAT activity; Increased acetylation of histone proteins at lysine 14 (H3K14); Reduced phosphorylation of tau protein	(Pan and Niles, 2015), (He et al., 2005), (Zhong et al., 2003), (Wang et al., 2021)
	Protein Kinase C, GSK-3β	Activation of protein kinase C phosphorylation; Inactivation of GSK-3β leading to reduction of tau hyperphosphorylation	(Medina et al., 2011), (Shi et al., 2018), (Majd et al., 2017)
Parkinson’s disease	α-synuclein	Increased DNA methylation of α-synuclein	(Pihlstrøm et al., 2015), (Ono et al., 2012), (Yang et al., 2019)
Type 1 Diabetes Mellitus	p100, Rel, NF-кB, p38 MAPK, p300, p52, COX-2, iNOS	Inhibition of p300 HAT activity; Reduced histone acetylation of p52; Reduced phosphorylation of p38 MAPK (pro-apoptotic factor)	(Deng et al., 2006), (Deng et al., 2003), (Shi et al., 2012)
Type 2 Diabetes Mellitus	p38 MAPK, p300, NF-кB, p52, COX-2, iNOS	Inhibition of p38 MAPK via reduction of phosphorylation; Blocking LPS induced inflammation	(Ruiz et al., 2018), (Saldeen et al., 2001), (Choi et al., 2018), (Xu et al., 2018)
Cancer			
Histone modification	p65, p50, IкBα, cyclin-D1, MMP-9, BCL-X, XIAI	Inhibition of translocation of p65 to the nucleus; Decreased IK Kinase enzyme phosphorylation	(Qin et al., 2012), (Ju et al., 2016), (Ling et al., 2012a), (Zou et al., 2018), (Gao et al., 2017)
Non-coding RNA	miR-155, ARID2, Akt, p27, cyclin-D, RB-protein, E2F-DP	Inhibition of miR-155; Suppression of Akt phosphorylation	(Liao et al., 2018), (Zhang et al., 2016), (Long et al., 2017), (Gu et al., 2017), (Chen et al., 2016a)
	miR-16-5p, TGF-β I/II, SMAD-3, p300, CBP	Increased miR-16-5p activity; Reduction in SMAD3	(Zhang et al., 2018a), (Zhu et al., 2018), (Ji et al., 2015), (Wang et al., 2018b)
Artificial light	SIRT1, CLOCK, BMAL1, PER-2	Blocking SIRT1 from binding to CLOCK; Inhibition of deacetylation of BMAL1	(Sánchez et al., 2018), (Asher et al., 2008), (Sun et al., 2017a)
Cardiovascular diseases			
Cardiac fibrosis	TGF-β, SMAD3, lncRNA MALAT1, miR-141	Downregulation of MALAT1 and knockdown of miR-141; Inhibition of HDACs activity via suppression of SMAD3 phosphorylation	(Yeung et al., 2015), (Crespo et al., 2015), (Che et al., 2020), (Wu et al., 2018)
Hypertension	miR-27, AGT, ACE	Decrease in ACE, AGT, AGTRA1; Suppression of RAAS	(Zou et al., 2020), (Tain et al., 2017), (Tain et al., 2016), (Ohashi et al., 2019a)
Heart failure	Angiotensin II, p65, NF-кB, MMP-2	Inhibition of p65 phosphorylation; Reduction in MMP-2 via ROS scavenging	(Zablocki and Sadoshima, 2013), (Kim et al., 2012), (Kong et al., 2017), (Glezeva et al., 2019), (Jacob-Ferreira et al., 2013)
Atherosclerosis	Sp-1, Akt, PH4α-1	Increased phosphorylation of Akt	(Li et al., 2019b), (Abrams and Andrew, 2002), (Chuang et al., 2013)
Cardiac senescence	lncRNA H19, miR-675, p53, p21	Reduction in p53, p21	(Su et al., 2021), (Cai et al., 2016), (Li et al., 2019c)

### 1.1 Neurodegenerative Disorders

For years, the aging brain has directly been linked with manifestations of neurodegenerative disorders such as Alzheimer’s disease (AD) and Parkinson's disease (PD). These were characterized by anomalies in the synthesis of proteins and a conglomeration of misfolded proteins as a result of altered signaling pathways by epigenetic manipulations (Shukla et al., 2019). These disorders otherwise associated with low levels of melatonin were significantly reversed when melatonin was administered and its receptors triggered (Bondy and Sharman, 2007).

#### 1.1.1 Alzheimer’s Disease

Histone deacetylation (HDACs), one of the many epigenetic mechanisms now identified as playing a significant role in long-term potential and memory formation has been linked with memory and cognitive processes in the hippocampus and prefrontal cortex in Alzheimer’s disease. An experiment conducted on a *Drosophila* model showed how both knockdown and overexpression of HDACs resulted in long-term memory impairment in a specific way (Fitzsimons et al., 2013). By targeting specific classes of HDACs in these areas of the brain, the progression of the disease can therefore be seized and corrected. Recently, HDAC inhibitors have been established in patients with AD as potential targets due to their wide scope of effects, ranging from decreased deposition of Amyloid β-peptides, an immune response triggered by microglia, reduced β-secretase cleavage, regulated levels of SIRT1, BDNF, ABCA7, REST, LTP deficits and decreased phosphorylation and acetylation of Tau (Wang et al., 2019a; Rusek et al., 2019). Moreover, deacetylase classes of HDAC4 and HDAC2 levels were significantly upregulated in areas of the hippocampus, prefrontal cortex, and the basal forebrain region of neuronal cultured cells, in AD mouse models, (Chen et al., 2021). In the CK-p25 AD mouse model, increased binding of HDAC2 to promoter regions of genes with critical roles in learning and memory and synaptic plasticity was accompanied by reduced acetylation levels of H2BK5, H3K14, H4K5, andsq1 H4K12, reduced RNA polymerase II binding, and reduced gene expression (Giusti-Rodríguez et al., 2011). Thus, increased HDAC2/4 levels may lead to impaired neurological synaptic function and cognitive decline, a well-characterized pathological feature of AD.

*De novo* methylation and DNA methyltransferases (DNMTs) are critical in neurological function and behavior (Bayraktar and Kreutz, 2018). Consistent patterns of DNA methylation of particular interests have been reported in the replicated evidence of hypermethylation at CpG sites in neuropathology-associated epigenetic variations (Monti et al., 2020). Studies done on human postmortem brain samples revealed increased levels of mitochondrial 5-methylcytosine in CpG sites in the D-loop region of the entorhinal cortex, possibly leading to the formation of neurofibrillary tangles and accumulation of β-amyloid peptides (Blanch et al., 2016). Earlier on, it had been established that the D-loop region regulates mitochondrial transcription and regulation (Asin-Cayuela and Gustafsson, 2007), possibly explaining mitochondrial epigenetic mechanisms in AD. Corresponding immunoprecipitation followed by sequencing (ChIP-seq) analyses have revealed changes in the promoter (H3K4me3) or enhancer (H3K27ac) marks that correlate with gene expression alterations, while few alterations in heterochromatin or polycomb regions have been found (H3K9me3 and H3K27me3, respectively) (Akkers et al., 2009). H4K16ac is a histone mark localized to both enhancers and promoters generally associated with active gene expression, which is drastically lost in the AD cortex (Berson et al., 2018), pointing to an inability to upregulate H4K16ac in the aged AD brain.

Compelling evidence demonstrates melatonin’s neuroprotective activity in several models of brain injuries, impaired learning, and memory functions in AD (Shukla et al., 2019). Its antioxidant property has been shown to offer protective effects in AD through scavenging of the reactive oxygen and nitrogen species thereby decreasing Amyloid β-induced toxicity, and also solving sleep disturbance (Cardinali et al., 2010). Reduced formation of Aβ due to significant inhibition of inflammatory cytokines and interleukins triggered by microglial activation resulting in neuroinflammation, has been reported in transgenic AD mice when melatonin was administered (Olcese et al., 2009).

Lipopolysaccharide (LPS) has been widely used in studies including but not limited to, impaired learning and cognitive functions which are related to neurodegenerative diseases such as Parkinsonism and Alzheimer’s. Zhao et al. (2017), reported hyperactivity in the enzymatic action of the Histone acetyltransferases (HAT) coupled with an increase in histone acetylation H3 pattern in the fetal/immature brain of a maternal administered LPS. These effects were brought to control by maternally administered melatonin (Domínguez Rubio et al., 2017). LPS administered during pregnancy exhibited a decrease in histone acetylation activity of H4K8 and H3K9 in the hippocampus region, which exacerbated the impairment of learning and memory during the midlife and senectitude periods of the mice offspring (Li et al., 2016a). Previous studies have shown that impaired memory and learning are associated with the downregulation of histone acetylation, such as H3K9 (Singh et al., 2015) and H4K8 (Yan et al., 2015). This, therefore, increases the possibilities of melatonin as a potential drug in the treatment of LPS-induced neurodegenerative disorders through the mechanism process involving an increase of histone acetylation activity.

Abnormal regulation in phosphorylation of histone proteins modification by the histone enzymes, leading to accumulation of highly phosphorylated tau protein aggregates has been linked with cell growth and activation of gene transcription resulting in a dozen of neurodegenerative diseases including AD (Rossetto et al., 2012). The kinase activity of melatonin-induced phosphorylation and enhanced histone acetylation of proteins CBP (CREB-binding protein) and p300 was observed in Akt and MAPK/ERK1/2 signaling pathways on human neuroblastoma brain cells (Pan and Niles, 2015). Kinase enzymes of the MAPK/ERK1/2 signaling pathway have been implicated in the phosphorylation of histone proteins at serine -28 and -10 (H3S28 and H3S10) (He et al., 2005) resulting in the promotion of acetylation of histones at K9 (H3K9) (Zhong et al., 2003) and acetylation at K14 (H3K14) possibly causing tau phosphorylation. Xian li et al. demonstrated how melatonin enhanced the HAT activity of CPB/p300 histone proteins in the MAPK/ERK1/2 signaling pathway on neuronal stem cells correcting the abnormal phosphorylation through acetylation of histone proteins at lysine 14 (H3K14) (Li et al., 2017a), thus reducing the hyperphosphorylated effects on tau. A selective HDAC6 inhibitor effectively inhibited tau hyperphosphorylation leading to neuroprotection and promotion of neuronal neurite outgrowth (Wang et al., 2021). Furthermore, a synthesized hybrid molecule of melatonin and HDAC6 inhibitor showed a remarkable AD-modifying property having significantly higher efficacy compared to a positive control of HDAC6 inhibitor (He et al., 2021).

Additionally, phosphorylation of H3K14 and H3K9 have been demonstrated to cause activation of transcriptional regulation (Sawicka and Seiser, 2012), and enhanced chromatin structure relaxation (Wu et al., 2008), features seen in tau pathology of neurodegenerative diseases. Elsewhere, a comparison of tau extracted from the human brain diagnosed with AD with a normal brain shows upregulated levels of GSK-3β with elevated tau phosphorylation in the brain of the former, suggesting GSK-3β has a principal role in the pathophysiology of AD (Medina et al., 2011). Through MT2 receptor stimulation, melatonin causes a cascade of downstream activation of protein kinase C (PKC), phosphorylation, and inactivation of GSK-3β leading to the reduction of tau hyperphosphorylation (Shi et al., 2018). Studies have depicted downregulated levels of GSK-3β in neuronal cells exposed to melatonin, possibly due to inactivation of GSK-3β and consequently depletion of phosphorylation of tau at threonine/serine residues by melatonin (Majd et al., 2017).

The generation of the amyloid precursor protein initiates the development of the pathogenic peptides of Aβ, in the AD brain. Immunohistochemical analysis of the hippocampus and the frontal cortex revealed remarkable reductions in amyloid-beta deposition as a result of long-term treatment of melatonin through ERK phosphorylation and activation of transcription factors including CREB which in turn upregulates the ADAM 10 transactivation, causing a reduction in the generation of amyloid- β peptides (Shukla et al., 2015).

Collectively, these results show that further exploration of the role of melatonin enhanced transcriptional activation of these genes by their complementary proteins, together with the establishment of the connection between phosphorylated protein kinases and histone phosphorylation could give a deeper understanding and shade more light on the fate of the neuronal cells in the context of gene transcription and modulation of chromatin state and the pursuit of design and development of neurological therapeutics.

#### 1.1.2 Parkinson's Disease

PD is a neurological disorder due to cytoplasmic aggregation of the α-synuclein protein within the neurons, jointly with the inadequate or loss of dopamine in the neurons in the regions of substantia nigra, resulting in motor and non-motor manifestations such as bradykinesia, tremor, rigidity, instability of the posture, random-eye movement, sleep behavioral disorder, cognitive impairments and autonomic dysfunction (Jankovic, 2008). Epigenetic mechanisms have been reported in PD as a vital factor in the etiology of this disease (Labbé et al., 2016). Alteration in the methylation process has been seen in CpG subset sites in PD patients. In one study, using 335 PD subjects, 82 CpG sites of DNA methylation were identified to be altered (Chuang et al., 2017). Another cohort study identified distinct patterns in methylation of CpG sites which was progressive as the disease advanced. The study also observed modulations in methylation when dopamine therapy was used (Henderson-Smith et al., 2019). Furthermore, the same study reported hypomethylation of α-synuclein which increased the expression of this protein possibly contributing to PD progression. The expression was however downregulated in levodopa exposure characterized by hypermethylation of α-synuclein, indicating that dopamine therapy may cause modulations in the methylation of this gene possibly controlling the progression of PD (Schmitt et al., 2015). Moreover, reduction in the action of sirtuin coupled with hyperacetylation of histone sites including H3K27, H3K9, and H2BK15 at the PD prefrontal cortex, have been profoundly dysregulated at genomic levels hence associated with the pathology of PD (Park et al., 2016). It was reported that disease-dependent elevation of histone acetylation seen in human PD could be due to degeneration of dopaminergic neurons and infiltrating activated microglia both contributing to the disease (Harrison et al., 2018) and that the use of HDACs inhibitors could offer a novel direction in the treatment approach of the PD (Sharma and Taliyan, 2015). Since their discovery, numerous miRNAs have been associated with the pathology of PD. As reviewed by SY Goh, miRNAs including miR-29, miR-485, let-7, miR-30, and miR-26 have recently been established as the miRNAs of interest in the pathogenesis of PD especially when their expression is dysregulation (Goh et al., 2019). For instance, miR-7 and miR-153 have been implicated in the pathology of PD where they have been identified to specifically bind to 3′-untranslated regions of α-synuclein, connotating that overexpression of these miRNAs can cause a significant decrease in the protein and mRNA levels of α-synuclein in patients with sporadic PD (Doxakis, 2010).

The biological effects of melatonin regarding PD are vastly widespread. Studies have demonstrated its neuroprotective effects in improving both motor and non-motor impairments experienced by PD patients (Medeiros et al., 2007). Melatonin alleviated oxidative stress involved in neurodegeneration possibly via increasing SOD, CAT, GPx, and through decreasing MDA and dopaminergic neuronal death in the SN of a rat model of PD (Ozsoy et al., 2015). Administration of melatonin was premised on the inhibition of autophagic-induced neuronal cell death and neurotoxicity in PD (Su et al., 2015). Similarly, neuroinflammation reported in PD caused by the cyclooxygenase type 2 pathway was decreased by melatonin (Ortiz et al., 2013). Pathologically, this supplement decreased the a-synuclein levels in the striatum in monkey models of PD and also protected against dopamine loss in the substantia nigra, and further replenished the striatal dopamine loss (Paul et al., 2018). Genetically, mutations of the leucine-rich repeat kinase 2 (LRRK2) gene associated with sleep disorders and PD were significantly decreased when melatonin was administered (Sun et al., 2016).

Epigenetic regulation of specific genes by HDACs through impairment of histone acetylation has been attributed to PD, accomplished through inhibitors of HDACs whose effects could lead to increased histone acetylation and transcription of genes that encode specific neurotrophic factors or via a reduction in the accumulation of neurotoxic proteins (such as α-synuclein) causing a neuroprotective effect (Shukla and Tekwani, 2020). A good example of this manifestation is depicted when HDAC inhibition by valproic acid demonstrated an upregulation of melatonin receptors and eventually reduced neurotoxic proteins thereby increasing the availability of melatonin hormone in the rat brain causing beneficial effects in neuronal related diseases (Bahna and Niles, 2017). Similarly, through inhibition of HDAC, valproic acid improved PD-like motor and non-motor symptoms in mice models (Kim et al., 2019) which eventually increased dopaminergic transmissions (Green et al., 2017). Although there are few studies on the epigenetic effects of melatonin on PD, melatonin functions on neuronal cells are undeniable great benefits, and further studies confirming its activities on HDACs via promotion of acetylation of histone proteins and prevention of dopamine loss in PD may prove vital.

DNA hypomethylation of the α-synuclein has been reported as a pathogenic substrate attributed to the development of Lewy bodies diseases such as PD. One such study, a cohort survey, showed how all PD subjects exhibited significant hypomethylation of the a-synuclein compared to the controls suggesting a possible nexus between a-synuclein, promoter hypomethylation, and the risk of PD (Pihlstrøm et al., 2015). Previously, melatonin has been used as a neuroprotective agent due to its anti-assembly effects against mutant α-synuclein-induced injury (Brito-Armas et al., 2013) and mitigation of neurotoxicity in the substantia nigra (Ono et al., 2012). Melatonin is well known for its powerful antioxidant effects by scavenging free radicals and also by preventing catecholamine auto-oxidation observed in PD via the O-methylation process, thereby preserving mitochondria and normal DNA methylation (Yang et al., 2019). Together, these findings suggest that melatonin’s beneficial effects in PD could occur through modulation of DNA methylation, and future research on this mechanism could prove vital.

## 2 Diabetes

### 2.1 Type 1 Diabetes

Type 1 diabetes (T1D) also known as insulin-dependent diabetes mellitus is an autoimmune condition characterized by inflammatory responses due to the influx of immune response cells in the pancreas, leading to the destruction of insulin-producing beta cells (Tai et al., 2016). Interaction between environmental and genetic factors has caused alteration of gene expression through epigenetic regulations in T1D (Cerna, 2019). Several studies point to the involvement of inflammatory inducers such as cytokines and lipopolysaccharides, which damage cellular and molecular components (Pestana et al., 2016) as among the causative agents of T1D. Cyclooxygenase-2 (COX-2) and inducible Nitric Oxide Synthase (iNOS) expressions have been vindicated in this inflammatory induced response (Chen et al., 2016b). These inflammatory inducible proteins have been reported as upstream factors of T1D via activation of transcription factors through binding of the kappa beta (NF-кB) promoter regions resulting in modulation of epigenetic chromatin remodeling (Hu et al., 2016a). Changes in DNA methylation were observed at the insulin region in the islets as a result of cross-talk between the β-cells affiliated with DNA methylation and the immune response which suggested that an increase in immune response such as cytokines, can result in an increase in DNA methylation and decrease in insulin levels, a phenomenon observed in T1D (Rui et al., 2016). Histone modification via hyperacetylation was implicated in T1D associated with COX-2 expression, whereby monocyte acetylated histone H4 levels were remarkably elevated in diabetic patients relative to non-diabetic patients (Chen et al., 2009). The same study furthermore highlighted how these hyperacetylations were restricted to patients who had no complications further suggesting the potential protective effect of histone hyperacetylation in T1D patients with complications such as endothelial and vascular dysfunctions (Chen et al., 2009).

Though inadequately studied, the association between miRNAs and immune cells has been established in T1D. Studies have found that dysregulation of expressed miRNAs which correlates with immune cells such as T cells and B lymphocytes was linked to the onset of T1D and that these miRNAs when manipulated to participate in the preservation of cells of the pancreas as reviewed by Mohammad Taheri et al. (2020).

**FIGURE 1:**
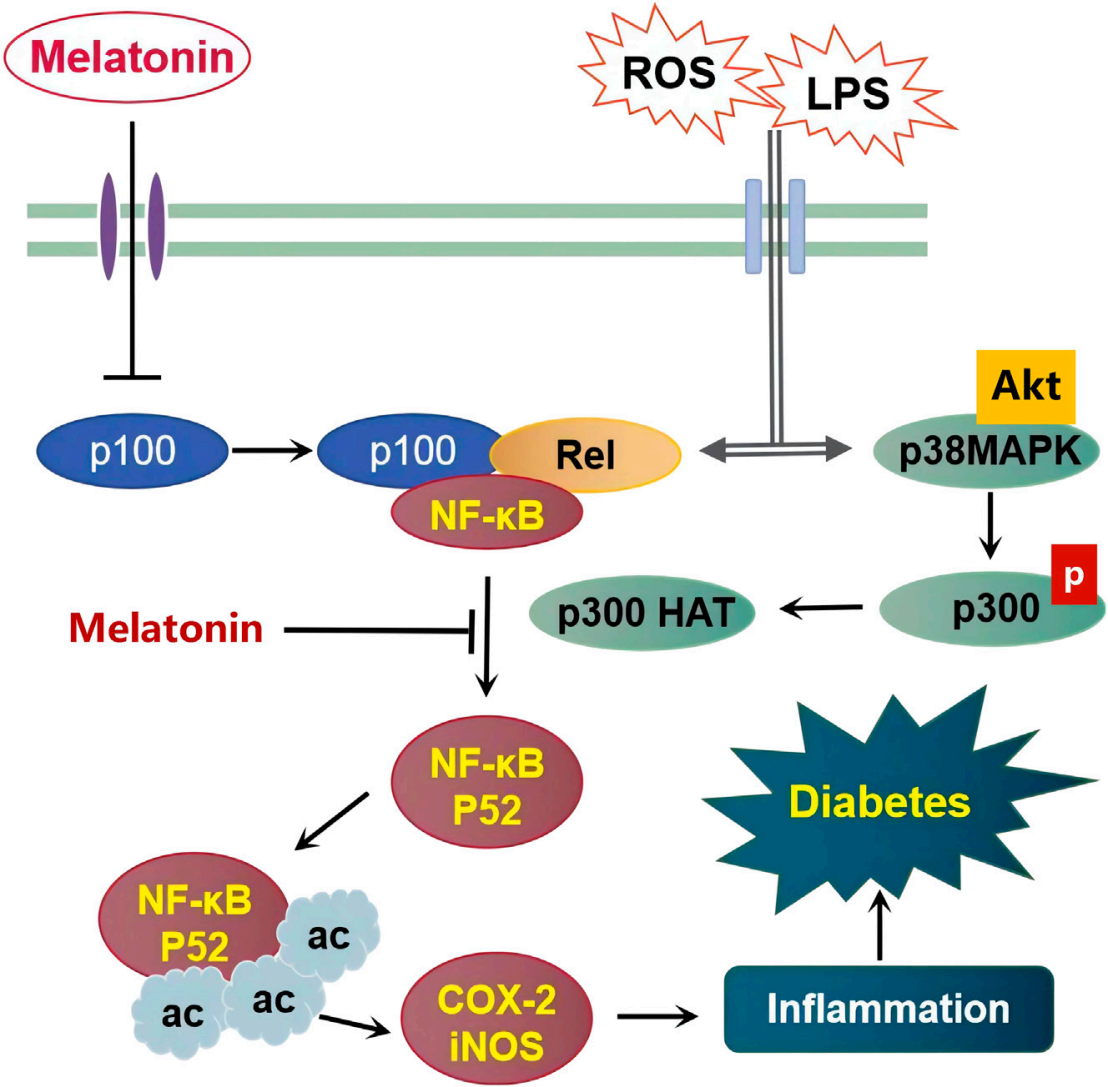
Schematic representation of the proposed mechanism underlying melatonin’s protective effects on diabetes caused by inflammatory inducers including Lipopolysaccharides (LPS) and Reactive Oxygen Species (ROS).

The importance of melatonin in immune cells has been established through modulation of responses of pancreatic cells whereby melatonin exerted beneficial effects in type 1 diabetes through suppression of T cell proliferation in the islets (Lin et al., 2009). Wu Guo Deng et al. demonstrated melatonin’s anti-inflammatory mechanism via suppression of the expression of pro-inflammatory genes COX-2 and iNOS through epigenetic modulation (Deng et al., 2006). They showed how melatonin can reduce the p52 binding to the chromatin of COX-2 and iNOS promoter regions through inhibition of p300 HAT activity (Figure 1). The study further established that increase in p300 HAT activity consequently increased histone acetylation of p52 which increased the expression of the pro-inflammatory mediators. Several lines of studies have explained the relationship between the action of p300 HAT and that of iNOS and COX-2, that is, a decrease in p300 HAT activity leads to a decrease in transcriptional activities of the iNOS and COX-2 and vice versa (Deng et al., 2003). The exact mechanism by which melatonin inhibits p300 HAT activity is still elusive. However, overexpression of p300 protein interacts with the promoter regions, enhancing the transcriptional signal which results in the optimization of pro-inflammatory activation (Shi et al., 2012). Usually, these inflammatory mediators are bound by the p52 domain due to p300 HAT activity (and eventually increased histone acetylation of p52), which results in the opening up of the chromatin structures, making them available to trans-activators hence enabling their transcription (Deng et al., 2006), intensifying inflammation. However, this process was inhibited by melatonin, suggesting its regulatory role in pro-inflammatory genes by transcriptional mechanisms (Shi et al., 2012). Kellogg et al. (2008) postulated that the COX-2 pathway may provide a potential therapeutic alternative in diabetic peripheral neuropathy. In normal conditions, p52 is a transcriptional repressor protein generated from p100 under a tightly controlled process (Vallabhapurapu et al., 2015). The nexus of how transcription is activated through the p100 route via epigenetic mechanism is still a mystery and further investigations may provide a better therapeutic strategy in the development of target-specific drugs.

### 2.2 Type 2 Diabetes

P300 has further been investigated in Type 2 diabetes through different epigenetic modulated mechanisms to that of T1D. P300 protein has been implicated in insulin secretion patterns in the beta cells of the pancreas. Bompada et al. (2016) illustrated how p300 knockout led to misshapen secretion of insulin (a typical feature of T2D) and apoptosis of beta cells of pancreases, marked by a decrease in p300 acetylation in presence of high glucose, and an increase in acetylation when an HDAC inhibitor was introduced. Melatonin has been shown to promote beta cell survival by preventing glucotoxicity-induced beta cells loss via p38 MAPK inhibition. In the pancreases, it has been reported that phosphorylation of Akt and/or p38 MAPK plays an important role in beta-cell survival (Wijesekara et al., 2010). On the other hand, diabetes is characterized by dysfunction of pancreatic beta cells and the development of apoptosis due to raised levels of glucose above normal (Butler et al., 2003). Moreover, glucotoxicity and other inflammation-inducing agents have been implicated in the phosphorylation of p300 due to the activation of p38 MAPK in the pancreatic beta cells, leading to cell degradation and apoptosis (Ruiz et al., 2018). However, this process was reversed as the activation of pro-apoptotic factor p38 MAPK was blocked when melatonin was administered (Saldeen et al., 2001).

Overwhelming evidence shows activation of p38 MAPK in insulin-releasing cells causes a damaging effect on the beta cells. Natalia Makeeva et al. (2006) reported that upon activation of p38 MAPK due to the high levels of cytokines like iNOS, p38 MAPK was maintained at a hyper-phosphorylated state, the beta cells producing insulin was driven into apoptosis and eventually cell death. Melatonin signaling was shown to alleviate induced-cell apoptosis and improved the deleterious effects caused by insulin resistance (Figure 1) due to glucotoxicity and oxidative stress in human beta cells (Costes et al., 2015), through inhibition of the apoptotic factor p38 MAPK (Park et al., 2014) and reduction of p38 MAPK phosphorylation (Choi et al., 2018). In addition, Xiongfei Xu et al. (2018) demonstrated melatonin’s ability to attenuate LPS-induced inflammation in macrophages through Akt phosphorylation signaling. Akt phosphorylation has been reported severally as a critical modulator of GLUT 4 translocation (an effector of glucose uptake in tissues) thereby enhancing the effects of insulin on target tissues (Choi et al., 2018).

## 3 Cancer

Global statistics today place cancer as one of the leading causes of death next only to heart-related diseases in the order of incidence rate (Sung et al., 2021). Evidence shows studies and extensive reviews on different types of malignant neoplasia inhibited or controlled by the effects imparted by melatonin administration both *in vivo* and *in vitro* studies (Bondy and Campbell, 2018; Favero et al., 2018). Prospectively, several mechanistic approaches have been suggested in the treatment of cancer. Due to melatonin’s broad spectrum of action, epigenetic modification has been one of the fundamental tools used to unravel the possible mechanisms involved by this drug in numerous cancer types. Melatonin has also been widely implicated as a complementing drug in cancers where it enhances and synergizes the chemotherapeutic effects of other drugs (Gao et al., 2017).

### 3.1 Histone Modifications

COX-2 is an iso-enzyme responsible for the formation of prostanoids such as prostaglandins and thromboxanes, whose overexpression has been reported not only in inflammatory diseases but in the induction of numerous types of cancer as well (Roelofs et al., 2014). Melatonin on the other hand has been reported severally as an inhibitor of COX-2 in cancer cells (Yi et al., 2014; Shrestha et al., 2017). Several explanations have been explicated including the epigenetic approach which involves the regulation of trans-activator and co-activator genes. For instance, the Factor Nuclear kappa B (NF-ĸB) is a transcriptional factor located in the cell cytoplasm functioning to regulate gene expressions involved in cell survival, proliferation, inflammatory responses, and metastasis (Taniguchi and Karin, 2018). In normal cells, NF-ĸB is usually in a resting/inactive state in the cytosol and its activation is often regulated to maintain the cell homeostasis, and activation of immune responses thereby protecting the cell from environmental factors that would otherwise destroy it (Liu et al., 2017). In cancer cells, NF-ĸB is constitutively activated due to aberrant protein expression or alterations in normal regulation by the regulatory proteins leading to mutation (Park and Hong, 2016).

**FIGURE 2:**
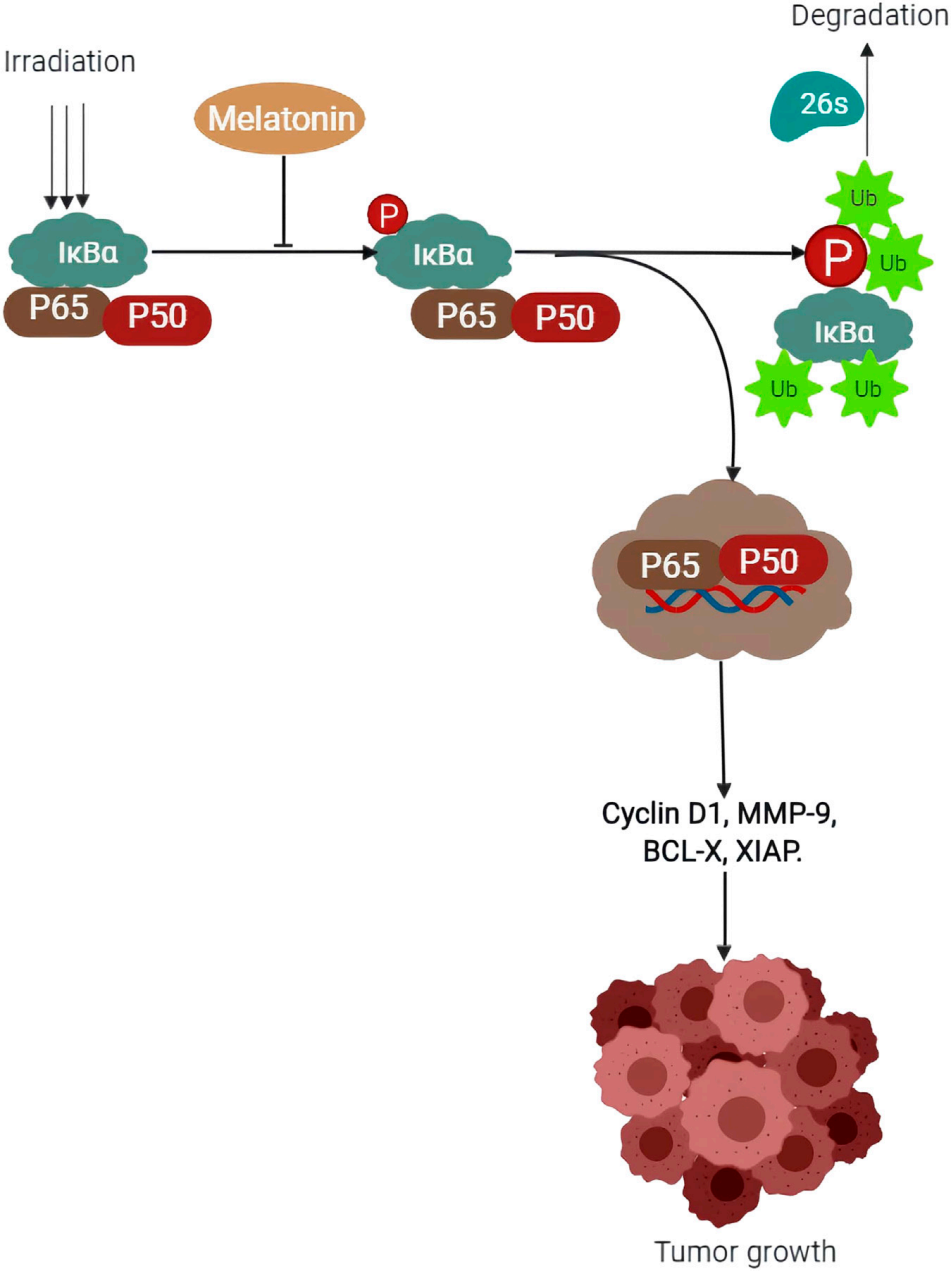
Schematic representation of the proposed mechanism on how melatonin prevents tumor growth by decreasing IK kinase enzyme (IkBα) phosphorylation.

NF-ĸB activation has been reviewed in the inhibition of apoptosis, and induction of tumorigenic enhancing genes such as MMP-9, Bcl-xl, XIAP, and cyclin D1, leading to metastasis (Fuchs, 2013). Through suppression of NF-ĸB (p65) gene expression (Figure 2), melatonin can inhibit its activation and subsequent translocation to the nucleus (Qin et al., 2012), preventing metastasis in cancer cells (Gao et al., 2017). Huaiqiang Ju et al. attributed this to melatonin’s ability to decrease NF-ĸB protein activity via phosphorylation. The inhibitory protein, IкBα which usually binds to NF-кB, causes the latter to migrate into the nucleus when the IK kinase enzyme phosphorylates IкBα and is subsequently degraded by proteasomal enzyme 26S via ubiquitination, leading to NF-ĸB translocation into the nucleus. They concluded that melatonin administration to pancreatic ductal adenocarcinoma cells led to a decrease in phosphorylation of IкBα which helped to explain the low levels of p65 protein in the nucleus of melatonin treated groups (Ju et al., 2016). Further evidence on pancreatic cancer cells shows that upon activation (as a result of abnormal activation of p65 binding with p50 protein), a heterodimer is formed which enters the nucleus to begin the process of transcription and eventually tumor development and progression (Ling et al., 2012b). Zhen Wei Zou et al. (2018), reported that activation of NF- ĸB signaling evidenced by elevated levels of phosphorylated p65 and other gene products that enhance the invasion of cancer cells, was eradicated upon treatment with melatonin. Additionally, melatonin showed synergistic effects to 5-fluorouracil (5-FU) enhancing its antitumor activity by facilitating the migration of p50/p65 from the nucleus to the cytoplasm, reducing NF-кB dependent cancer development and progression (Gao et al., 2017). It is therefore evident that inhibition of the NF-ĸB pathway by targeting p65 protein could be a breakthrough in the discovery of drug therapy for various types of cancer. Melatonin offers a promising and noble target in this perspective, as far as drug efficacy and safety are concerned.

### 3.2 Non-coding RNA

In the treatment of cancer, the actual mechanism underlying the association between miRNAs and melatonin remains elusory. In recent times, there has been a surge in the involvement of miRNAs in the advancement of tumors which has attracted attention in the field of research. These miRNAs have been demonstrated to regulate the expression of genes by inactivating messenger RNA by either preventing ribosomal binding or cutting the mRNA which is further destroyed by the cell leading to less or no translation of mRNA, a process resulting in gene silencing (Hammond et al., 2001). The study on miR-152 has been on the rise due to its tumor-suppressing activity. Constant overexpression of miR-152 in cancer cell lines caused a remarkable decline in cell proliferation and angiogenesis (Li et al., 2016b). Sun et al. (2017b) showed that miR-152-3p inhibited invasion and development of glioblastoma cells by down expressing DNA methyltransferase thereby hindering hypermethylation of neurofibromatosis 2 (NF2). Treatment with melatonin showed elevated levels of miR-152-3p in breast cancer cells when compared to control groups (Marques et al., 2018), suggesting melatonin’s role in cancer suppression.

Another miRNA, miR-483 overexpression has been recently shown to play an oncogenic role in thyroid cancer (Zhang et al., 2019), breast cancer (Huang and Lyu, 2018), colorectal cancer (Liang et al., 2019), esophageal squamous cell carcinoma (Ma et al., 2016), ovarian cancer (Arrighetti et al., 2016), hepatocellular (Lupini et al., 2016), and pancreatic ductal adenocarcinoma (Wang et al., 2015). Earlier on, Clokie et al. (2012) had shown how the addition of miR-483-3p antagonist led to an increase in the synthesis of melatonin by the pineal gland and eventual attenuation of tumor invasion and progression. Thus, inhibition of miR-483-3p and subsequent elevation of melatonin present a promising tumor therapeutic target.

MiR-155 has been a gene of interest implicated in the development of cancer, just a while ago. Studies performed on hepatocellular carcinoma showed elevated levels of miR-155 which acted as an oncogene thereby promoting cell proliferation, progression of the cell cycle, and inhibition of apoptosis (Liao et al., 2018). Zhang et al. (2016) explained that this phenomenon was a result of the downregulation of ARID2, a tumor suppressor gene, which subsequently activated the Akt phosphorylation signaling pathway leading to cancer progression. Accruing evidence shows melatonin’s prowess in arresting cell cycle progression in hepatocellular carcinoma by downregulation of p-Akt dependent pathway (Long et al., 2017). Activation of the Akt pathway has been shown to cause oncogenic transcription factors to bind and inactivate phosphorylated Rb protein leading to the transcription factor E2 promoter-binding-protein-dimerization partner protein (E2F-DP) release, pushing the cell into the synthesis phase, a process that is reversed by melatonin (Long et al., 2017). On the same note, melatonin has been reported to inhibit miR-155 in glioma (Gu et al., 2017) and glioblastoma cells through suppression of Akt phosphorylation, inhibiting its epigenetic modified oncogenic transcriptional activity (Chen et al., 2016a).

**FIGURE. 3:**
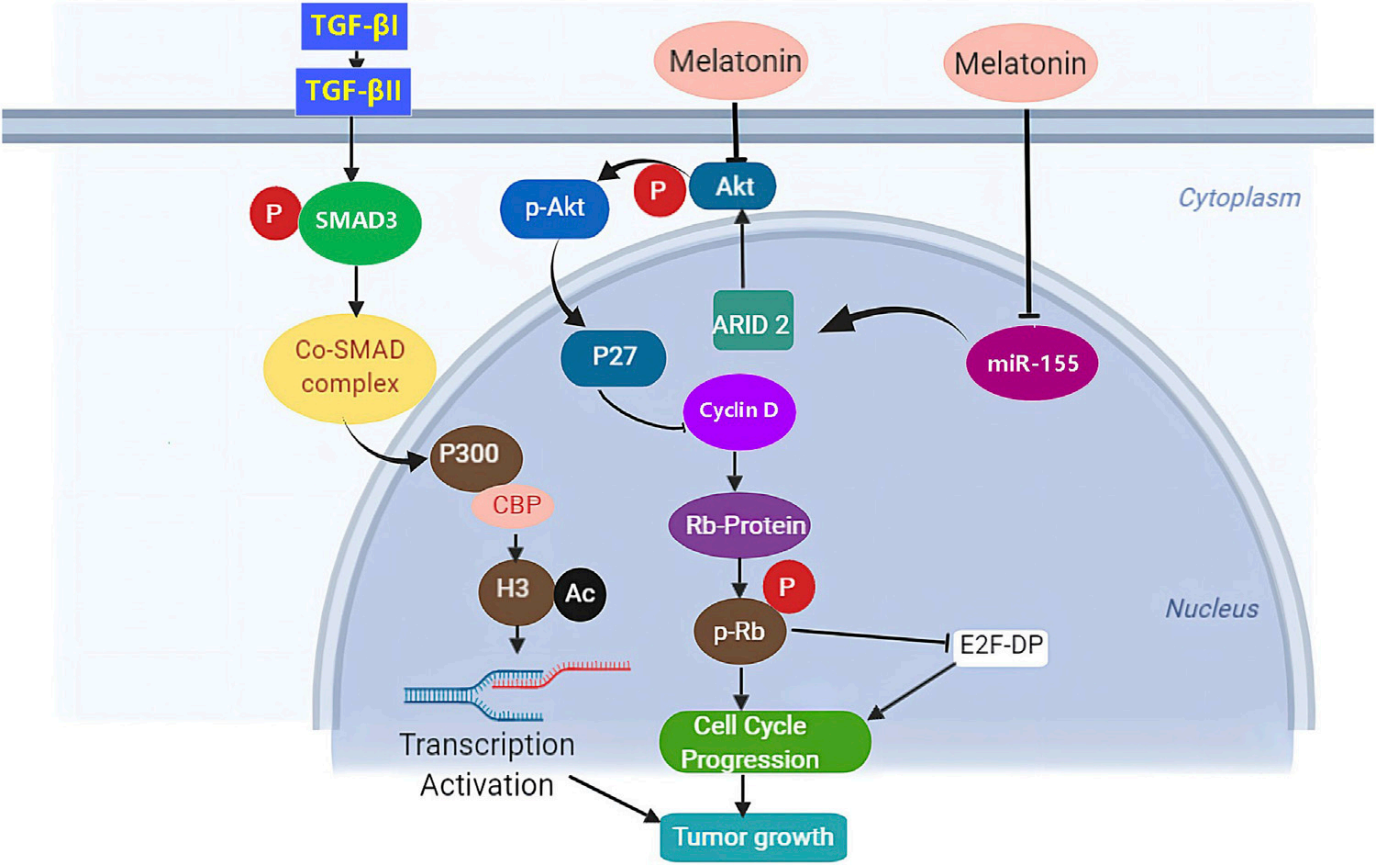
The proposed mechanism underlying the anti-tumor effects of melatonin. Melatonin arrests cell cycle progression through downregulation of phosphorylated protein kinase B (p-Akt) dependent pathway.

Studies pointed towards miR-16-5p are the latest in trying to explain the epigenetic mechanism in cancer by the non-coding RNA molecules that regulate gene translation in various types of cancers. Studies reveal that aberrant expression of miR-16-5p resulted in malignancies including gastric (Zhang et al., 2015), (Krell et al., 2018), breast (Rinnerthaler et al., 2016), (Zhang et al., 2018a), bone (Sang et al., 2017), ovarian (Dwivedi et al., 2016), hepatic (Wu et al., 2015), prostate (Wang et al., 2019b), cervical (Wang et al., 2008), and lymphatic system (Calin et al., 2002). Treatment with melatonin suppressed gastric cancer (Figure 3) via upregulation of miR-16-5p expression and downregulation of SMAD3 (Zhu et al., 2018), indicating that the TGF-β/SMAD pathway might be a possible molecular mechanism by which melatonin precludes cancer progression. TGF-β is a protein known to control cell functions including cell growth, proliferation, and apoptosis (Lustri et al., 2017). Pathologically, the TGF-β/SMAD signaling pathway has been associated with migration, invasion, and metastasis (Ji et al., 2015). Here, the TGF-βI trans-phosphorylates type II (TGF-βII) induces phosphorylation of SMAD3, consequently activating the formation of a complex with SMAD4 which migrates and accumulates in the nucleus where they interact with transcription cofactors and promoters which in turn regulates transcription of target genes, eventually enhancing cell proliferation and metastasis (Ji et al., 2015). Melatonin exposure significantly attenuated gastric cancer by enhancing miR-16-5p expression which targeted SMAD3 and resulted in negative regulation of its abundance (Zhu et al., 2018). Elsewhere, melatonin was reported to alleviate liver fibrosis through TGF-β1/SMAD signaling pathway (Wang et al., 2018b). Hongliang Jiang et al. further demonstrated that miR-16-5p played a tumor suppressor role via downregulation of SMAD3 (Zhang et al., 2018a). How melatonin interacts with miR-16-5p to impart its cancer-suppressing activity still needs further clarification and future studies to confirm the molecular mechanism are vital. This approach puts melatonin and miR-16-5p/TGF-β/SMAD pathway as a possible candidate for therapeutic target and possibly understanding of the etiology of different types of cancer.

### 3.3 Artificial Light and Histone Modification

Epidemiological studies show a strong association between artificial light and the prevalence of cancer (Garcia-Saenz et al., 2018). Modern artificial lights especially those with blue light emissions have been reported to cause a litany of health problems with cancer being among them (Kamat et al., 2016). The suprachiasmatic nucleus (SCN) functions to regulate the circadian cycle which is controlled by the external environment (day and night clock). Several reviews have been put forth trying to explain how environmental factors such as artificial light can cause circadian disruptions by causing desynchronization of the circadian pacemaker (SCN), resulting in decreased levels of melatonin secretions from the pineal gland (Benke and Benke, 2012). Concerns have been raised regarding the effects of the artificial light at night associated with hormonal imbalances (melatonin) and impaired circadian rhythms (Russart and Nelson, 2018), causing a myriad of diseases including cancer. Studies show that melatonin attenuates the effects of breast, prostate, liver, and skin cancers associated with ill-timed light exposures (early morning, late evening, night) (Dauchy et al., 2018). In the recent past, epigenetic modification affecting the circadian rhythm has been used to explain melatonin’s mechanism of action. Through the activation of the MT1 receptor, melatonin was able to prevent invasion and metastasis of breast cancer (Hill et al., 2015). Agbaria et al. (2019) demonstrated that, when artificial light at night (ALAN) with increased irradiances was administered in rats’ breast cancer, melatonin levels were significantly decreased, while the levels remained normal in the control group. This had been confirmed earlier by Sánchez et al. (2018) who implicated that melatonin resynchronized dysregulated circadian rhythm by preventing SIRT1 from binding to CLOCK/BMAL1 dimer which would otherwise cause deacetylation of BMAL1 resulting in less transcription and degradation of the period (PER) proteins. Earlier, SIRT1 had been implicated in the regulation of the circadian cycle through the deacetylation of PER2 protein (Asher et al., 2008). The CLOCK protein has been reported to exhibit histone acetylation activity necessary in the modulation of circadian rhythm in cancer animal models (Sun et al., 2017a).

It was also found that melatonin upregulated Casein Kinase 1 (CK1) protein levels, increasing the binding sites available for phosphorylation to the transcription factors (Sánchez et al., 2018). These findings together support melatonin’s effects on hepatocellular cells which might also have contributed to its protective activity through modulation of the circadian rhythm.

## 4 Cardiovascular Diseases

### 4.1 Cardiac Fibrosis

Cardiac fibrosis is a common manifestation of chronically stressed myocardial tissues characterized by abnormal thickening of the valves of the heart due to the proliferation of the fibroblasts (Kong et al., 2014). The functioning heart muscles are replaced by the thick dysfunctional fibrotic tissues resulting in heart failure and sometimes death. Currently, though there exists no convincing therapy that can prevent the onset and progression of this condition, understanding the pathophysiology of this process may prove crucial and beneficial.

Strides have been made in the interest of employing epigenetic therapies to arrest myocardial fibrotic degeneration through stimulation of human cardiac fibroblasts to suppress or express pro-fibrotic protein genes which have the effect of modulating DNA-methylating genes such as DNMT1 and DNMT3a/3b (Yu and Xu, 2015). Activated myocardial fibroblasts in the rat model resulted in an increase in DNMT3a which in turn led to overexpression of ERK1/2 coupled with suppression of RASAL1 (a Ras-GTPase-activating protein) genes, causing fibroblast activation and cardiac fibrosis (Grimaldi et al., 2017). A DNA methylation inhibitor 5-azacytidine has been reported in the sumoylation of interferon regulatory factor-1 (IRF-1) causing improvements in cardiac fibrosis (Jeong et al., 2015).

The protective effects of melatonin have been previously investigated, effectively alleviating cardiac fibroblast-induced cardiac fibrosis via inhibition of mitochondrial oxidative injury and regulation of SIRT3-mediated SOD2 deacetylation (Jiang et al., 2020). Melatonin also exerted anti‐fibrosis effects in all phases of fibrotic pathogenesis including inhibition of epithelial cell injury, blockading of inflammatory cytokine release, reduction in activation and proliferation of fibrogenic effector cells, and decrease in deposition of ECM (GAG and collagen I and III) (Hu et al., 2016b).

Big strides have been made in understanding melatonin-induced epigenetics on cardiac fibrosis and towards the identification of therapeutic targets. One such study conducted by Crespo et al. (2015) postulated that the development of fibrosis greatly depended on the pro-fibrogenic cytokine TGF- β, directly linked with hyperphosphorylation of SMAD3. TGF-β1/SMAD pathway has been associated with fibrosis in the kidney (Wang et al., 2016), heart (Bansal et al., 2017) liver (Lee et al., 2018b) skin (Qin et al., 2018), and lungs (Jia et al., 2019). They demonstrated that through downregulation of TGF-β and subsequent decrease in phosphorylation of SMAD3, melatonin abrogated the transcription of target genes induced by the TGF-β1/SMAD signaling pathway (Crespo et al., 2015). TGF-β is a well-known collagen synthesis enhancer (Qin et al., 2018). Additionally, melatonin significantly attenuated the fibrosis process by suppressing the activation of TGF-β1/SMAD signaling in heart (Yeung et al., 2015), which resulted in the reduction of collagen deposition in the heart tissues. Similarly, melatonin produced anti-fibrotic effects through activation of TGF-β1/SMAD signaling pathways as a result of down-regulation of lncRNA MALAT1 and knock-down of miR-141 (Che et al., 2020). Elsewhere, the elevated expression of histone deacetylases in cardiac fibrosis in juveniles was attributed to decreasing melatonin levels resulting in heart failure, a condition that was reversed when external melatonin was administered and playing an inhibitory role to the HDACs. Earlier on, Wu et al. (2018) had postulated that melatonin could prevent programmed hypertension by inhibiting HDACs (Wu et al., 2014). However, the underlying molecular mechanism behind this inhibition needs further elucidation.

### 4.2 Hypertension

The in-depth knowledge of the renal-angiotensin-aldosterone system (RAAS) alongside *in vivo* and *in vitro* studies have suggested possible epigenomic-related mechanisms underlying the etiology of hypertension. Unusual activation of RAAS by the associated genes has been previously reported to cause an increase in blood pressure (Crowley et al., 2006). Angiotensinogen (AGT) and angiotensin-converting enzyme (ACE) have been reported as the main genes which code to the angiotensinogen protein and angiotensin-converting enzyme respectively, regulating the RAAS pathway and eventually blood pressure (Pei et al., 2015). Moreover, activation of RAAS genes due to impaired functioning of the RAAS system has been associated with pathophysiological conditions including essential hypertension. One such gene 11-Betahydroxysteroid dehydrogenase type-2 (HSD11B2), performed in a rat model demonstrated a positive association between hypermethylation and hypertension (Baserga et al., 2010). Similar findings were reported in newborn babies whereby hypermethylation of the (HSD11B2) promoter gene was linked to a high risk of essential hypertension (Zhao et al., 2014). Elsewhere, hypomethylation of promoter gene coding AGT in rat adipose tissues (Wang et al., 2014) and hypomethylation of ACE during pregnancy (Goyal et al., 2010) both led to the occurrence of hypertension. Studies on non-coding RNA demonstrated repressed levels of AGT and elevated expression of ACE in hypertensive individuals (Litwin et al., 2013). MiR-27a/b and 21 have been linked with the expression of RAAS-regulated AGT and ACE genes respectively, both leading to hypertension (Zou et al., 2020).

Melatonin has been implicated in the epigenomic regulation of hypertension by decreasing the mRNA expression levels of agt1, ace, and agtr1 which promoted vasodilation and alleviating hypertension (Tain et al., 2017). Tain et al. (2016), demonstrated that melatonin could decrease renal levels of *ace2, agt,* and *agtra1* genes. Additionally, melatonin attenuated hypertension through suppression of the renin-angiotensin system (Ohashi et al., 2019b). Recently, it has been reported and reviewed by several researchers, that aberrant methylation patterns of ACE and AGTR-1 promoter regions might be one of the underlying epigenetic mechanisms behind hypertension (Pei et al., 2015).

The non-coding RNAs could also play a role in the development and progression of hypertension through epigenetic modulations. Melatonin has been shown to regulate these non-coding RNAs thereby modulating the occurrence or progression of hypertension. Real-time PCR and western blotting results clearly illustrate how the overexpression of H19 and miR-675-3p coupled with downregulation of miR-200a in melatonin-treated rats (previously induced with hypertension) led to an increase in the expression of PDCD4 and a decrease in expression of IGFR1 proteins (Wang et al., 2018c). Changes in these two proteins eventually can result in apoptosis and suppression of smooth muscles of the pulmonary artery, a condition associated with the alleviation of hypertension (Liu et al., 2010). DNA-methylation induced by a methyl-donor diet resulted in programmed hypertension in women during the pregnancy period by altering renal transcriptomes, a condition that was reversed when maternal melatonin therapy was conducted. It was then reported that melatonin therapy could offer protective activity in adult male offspring against programmed hypertension mechanistically by regulating DNA-methylation (Tain et al., 2018) or playing a HDACs inhibitor role. To date, the actual nexus between melatonin and epigenetics in the pathophysiology of hypertension is still unknown. A deeper understanding of how the non-coding RNAs and DNA-methylation stimulates the RAAS, AGT, and ACE genes of the renal system leading to the development of hypertension, would be a step closer to better understanding the actual mechanism by which melatonin works to alleviate hypertension, making it easier to design and develop melatonin-based therapies.

### 4.3 Heart Failure

MiR-200 family is a target molecule in the pathology of heart failure. Liu et al. (2018) premised that upregulation of miR-200a due to increased secretion of protein CTRP3 (a paralog of adiponectin) in melatonin treated groups, led to activation of Nrf-2 and eventually abrogation of obesity-related heart failure. Contrarily, it was reported elsewhere that melatonin downregulated the expression of miR-200a which subsequently increased the apoptotic genes PDCD4 resulting in amelioration of pulmonary artery hypertension (Wang et al., 2018c). MiR-200 has also been implicated in arrhythmias (Daimi et al., 2015) and hypercholesterolemia associated with atherosclerosis (Martino et al., 2015).

**FIGURE 4:**
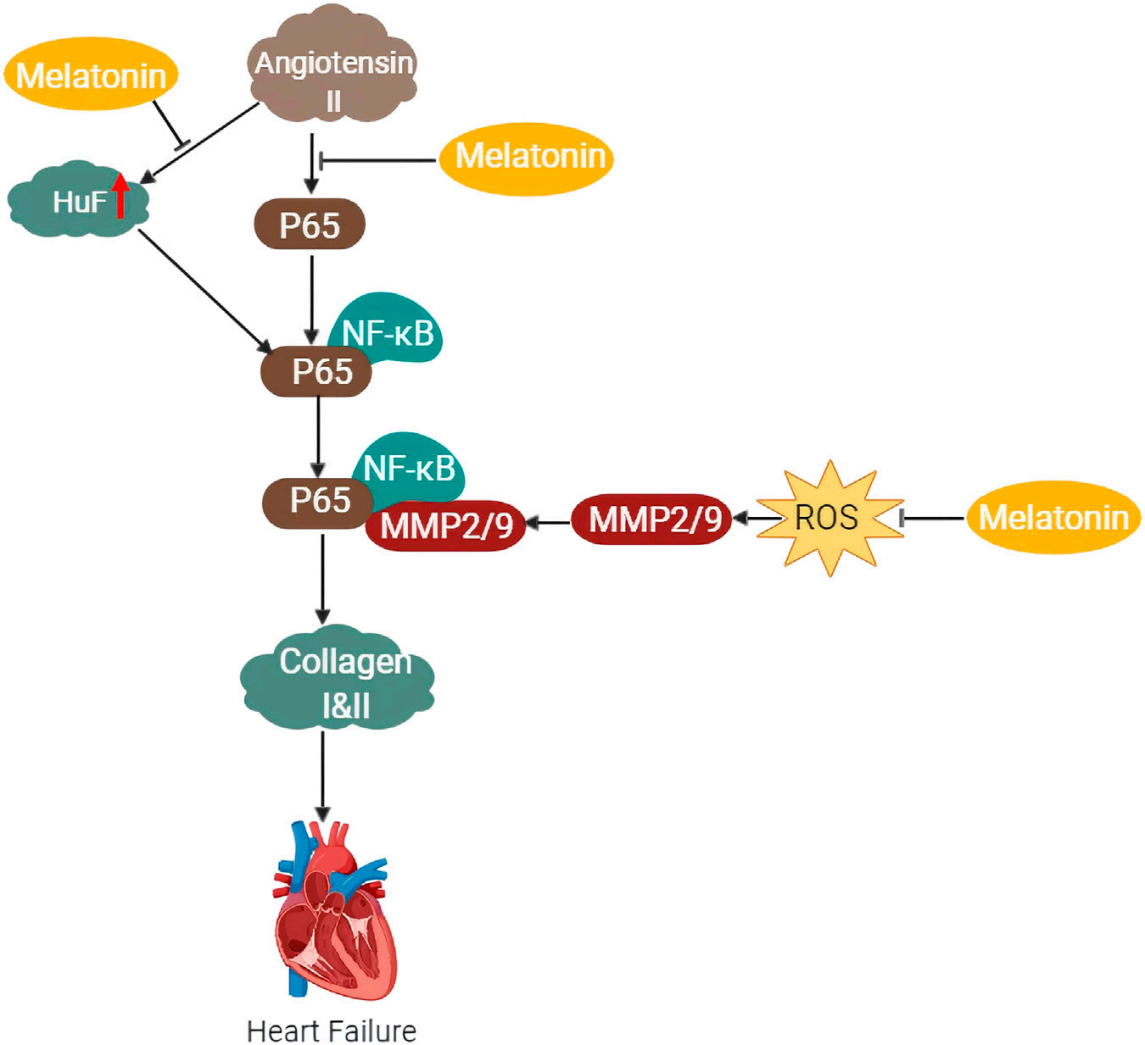
Schematic diagram of the proposed mechanism of the protective effects of melatonin against heart failure via inhibition of NF-kB and MMP2/9 pathways.

Angiotensin II a well-known pro-inflammatory regulator has been implicated in cardiac dysfunction of a failing heart through its association with reactive oxygen species (ROS) (Zablocki and Sadoshima, 2013) and phosphorylation of p65/RelA when overly expressed as seen in heart failure (Kim et al., 2012). The result is an activated p65 which binds to the promoter site of NF-кB in the human antigen R (HuR) (Kong et al., 2017) eliciting a binding activation to the MMP2, leading to collagen degradation and eventual heart failure. An increase in hypomethylation of MMP2 has been previously reported in blood samples and cardiac tissues of heart failure patients (Glezeva et al., 2019). Additionally, the multifaceted sites of MMP2 available for phosphorylation contain NF-кB sites in the promoter domains which have been previously demonstrated to cause modulation in its activities (Sariahmetoglu et al., 2007; Jacob-Ferreira et al., 2013). Melatonin on the other hand has been shown to downregulate MMP2 protein expression (Figure 4) through inhibition of the NF-кB signaling pathway and via ROS scavenging (Kong et al., 2017). Although post-translational modulations such as phosphorylation can regulate MMP2 expression, melatonin involvement in this regulation is yet to be demystified.

### 4.4 Atherosclerosis

Atherosclerosis is a condition characterized by the buildup of plaques from cholesterol, deposited on the inner walls of the endothelium, causing stenosis and heart attack, and sometimes death (Insull, 2009). Endothelium dysfunction due to degeneration causes the formation of these plaques which upon rupture have been reported to cause sudden coronary death, myocardial infarction, and heart attack (Hsiao et al., 2015). Therefore, plaque stabilization is an important approach in the pharmacotherapy of heart diseases stemming from atherosclerotic plaque. Changing the morphology and content of the atherosclerotic plaque could help alter the function of the plaque in the endothelium by either preventing or reducing its stringent effects when it ruptures (Libby and Aikawa, 2003).

Increasing the fibrous content through collagen synthesis is the basic idea of stabilizing atherosclerotic plaque. Recently, Li et al. (2019b) demonstrated this phenomenon by using the smooth muscle cells from ApoE^−/−^ mice. They found out that at high doses, melatonin was able to cause significant stabilization of atherosclerotic plaque. They further explained this occurrence to be caused by melatonin-induced Akt phosphorylation of the specificity protein 1 (Sp1), which cleaved to the promoter site and elevated the expression of the PH4alpha-1 gene. The PH4alpha-1 gene has been associated with the formation of a major component of the extracellular matrix known as 4- hydroxyproline in collagens (Abrams and Andrew, 2002). Sp1 is a ubiquitously expressed transcription factor illustrated to be activated through Akt phosphorylation (Chuang et al., 2013). Earlier on, Sp1 was shown to cause transactivation of the VEGF promoter region via Akt phosphorylation (Pore et al., 2004). Additionally, the pineal hormone has been implicated in the downregulation of MMPs and interstitial increase in collagen, leading to stabilization of the atherosclerotic plaque, as a result of elevated levels of fibrous tissues (Aikawa et al., 1998). An increase in MMPs has been widely reported to degrade and suppress collagen synthesis (Bertelsen et al., 2018). This reduction of collagen due to degradation can lead to the weakening of the plaque, inevitably causing its rupture (Braganza and Bennett, 2001). Computational chemistry studies showed that melatonin could inhibit the enzymatic activity of MMP-9 by docking on its active site preventing further action of the enzyme (Rudra et al., 2013). This pineal hormone has also been premised to cause an upswing in collagen synthesis in the experimental model (Drobnik et al., 2008), which could unwind the process of plaque rupture.

Different experimental reports have deduced molecular pathways involving inflammatory processes leading to atherosclerosis (Moriya, 2019). The role of miR-223 in the regulation of atherosclerosis has been studied in the recent past with a growing body of evidence reporting exciting findings on the association of miR-223 with inflammation-induced atherosclerosis (You et al., 2022). Tang et al. (2015) explained that through suppression of TLR4 and its subsequent downstream gene NF-кB, they managed to cease inflammation-induced atherosclerotic plaque. Lately, miR-223 has been linked to the inhibition of inflammatory processes related to endothelium dysfunction in atherosclerosis (Cavallari et al., 2019). The expression of tissue factor (TF) also known as thromboplastin was reportedly suppressed by miR-223, suggesting a possible anti-thrombogenesis mechanism in the formation of atherosclerotic plaque (Li et al., 2014). We have previously reported the progression of atherosclerosis through the pyroptotic process via lncRNA-MEG3/miR-223/NLRP3 pathway. Any attempts of knocking down miR-223 led to the blocking of melatonin’s inhibitory effects on pyroptosis, suggesting that there is a nexus between miR-223 and melatonin in the alleviation of atherosclerotic plaque (Zhang et al., 2018b). Although it is not yet defined how melatonin directly collaborates with miR-223 to impact anti-atherosclerosis action, the recent research findings on melatonin seem to corroborate its putative actions against atherosclerotic plaque.

### 4.5 Cardiac Senescence

The concept of aging and growing old seems not to go down well with many people; some people believe once they grow old, they might lose their worth, good looks, or importance in society. For a long period, several factors majorly environmental stimuli had been attributed to aging. In recent decades, scientists have been able to demonstrate that aging is hugely dependent and associated with genetic and epigenetic pathways which influence lifespan. Senescence involves a prolonged condition requiring the adaptation of the cell to a stressful microenvironment initiated by a deep epigenetic reconditioning response resulting in irreparable DNA damage. For instance, when mitochondrial DNA methylation was examined during the senescence period, a study concluded that hypomethylation of the mitochondrial CpG region was directly linked to cellular senescence as a result of oxidative stress (Yu et al., 2017). Likewise, the histone deacetylase inhibitor enzymes were intensely involved in the promotion of cellular senescence as a result of the upregulation of p16 and p21 gene expression via potent stimulation of cardiac fibroblasts (Tao et al., 2016). MiRNAs have also been involved in the physiological process of cellular senescence and aging. Through the TGF-β signaling pathway, miR-29 negatively regulates H4K20me3, an epigenetic target that leads to the progression of senescence (Lyu et al., 2018). These epigenetic alterations could be identified as targets for epigenetic therapies, from suppression of senescence-inducing triggers to positive activation of signaling pathways associated with anti-senescence.

Melatonin has been used substantially in many vital life processes, and its secretion and circulation decline gradually over time. For instance, Lahiri postulated that the levels of melatonin in the serum decreased significantly with age, in body tissues including the heart (Lahiri et al., 2004), and that chronic treatment with melatonin exhibited anti-senescence effects and also reduced deteriorative and oxidative changes associated with cardiac aging (Guo et al., 2017). Although a relatively new area of research, the role played by miRNAs in aging has gained interest among researchers in an attempt to prolong human life. Studies on these miRNAs associated with cardiac senescence and aging have shed much light on prospects, as to how they can be regulated to slow down cardiac senescence processes. LncRNA H19 has recently been implicated in the promotion of cardiomyocytes senescence by regulating the aging markers p53 and p21 (Zhuang et al., 2021). The lncRNA H19 silenced the MALAT1 and MIAT in fibrosis-related cardiac hypertrophy resulting in increasing in senescence protein markers p53 and p21 (Su et al., 2021). Melatonin on the hand actively produced antifibrotic action in cardiac fibrosis through inhibition of MALAT1 (Che et al., 2020). Additionally, H19-derived miR-675 has been implicated as a regulator of cellular senescence in aging-induced vascular dysfunction (Han et al., 2019). MiR-675 overexpression exhibited anti-senescence activity in cardiac progenitor cells in presence of melatonin through regulation of transcriptional factors p53 and p21 (Cai et al., 2016). Further, accumulation of p53 and p21 proteins have been reported in cardiovascular studies as promulgators of senescence through p53/p21 DNA-induced damage and cell cycle arrest rendering the cells senescence (Li et al., 2019c), a function that was reversed by exposure to melatonin (Cai et al., 2016). Altogether, these findings provide basic evidence of melatonin’s cardio-protective action, especially in an aging state.

## 5 Discussion

Melatonin has a broad spectrum of functions that are not well understood due to the vast effects mediated by its receptors MT1 and MT2. The expression of these receptors in different peripheral body cells insinuates that melatonin may associate and affect cellular functions ubiquitously in a fashion related to circadian rhythm. The characteristic rhythm of secretion of melatonin by the pineal gland is known to decline in an aged and/or diseased state (Hardeland, 2012). Although the actual mechanism by which melatonin and its receptors are aberrantly expressed is unknown, increasing evidence suggests that epigenetic modifications influence the development and progression of several diseases, such as neurodegenerative diseases, cancers, dysfunctional metabolic conditions such as diabetes, and cardiovascular diseases. For instance, melatonin receptors and eventually their signaling was significantly upregulated by the HDAC inhibitors via histone acetylation (Bahna and Niles, 2018), presenting a novel therapeutic strategy in counteracting negative gene transcription that might lead to age-related and/or disease condition as a result of impaired melatonin receptors. Despite this, the strategy is still inadequate since it is unclear which of the melatonin receptors is associated with the histone process. This, therefore, calls for strict discrimination and selection of specific receptor subtypes as targets and matching them with individual epigenetic isoforms of the histone process in an attempt to formulate personalized therapies. Furthermore, the susceptibility and possibility of pathological occurrence are linked with the interaction between genetic polymorphism and melatonin expression. The melatonin receptor B1 (MTNR1B) gene, a genetic polymorph, was implicated in T2D as an insulin controller, by regulating its secretion by beta cells of the pancreas (Prokopenko et al., 2009). This, therefore, suggests, that further information collected on gene polymorphism and the variants associated with controlled hyperglycemia in presence of melatonin could help develop therapies for patients with glucose disturbance.

Despite the well-collated information on the benefits of melatonin, there are inadequacies due to poor investigations on the subject. The nexus between melatonin and melatonin receptors in specific organs is not clearly explained. This is to say, the signaling routes by which melatonin regulates the epigenetic process, require further investigation. Secondly, we could not decipher how the action of melatonin affects different cells. For instance, in cancer, melatonin is seen doing the exact opposite of what is known of non-cancer cells (Li et al., 2017b). It is well illustrated that melatonin significantly declines in epigenetic regulated diseases (Bahna and Niles, 2018), and attempting to assess whether, this reduction has etiological significance or whether it is simply a basal epiphenomenon elementary to different disorders, is still a mystery. Hence, the need for more investigations in these areas to avoid unnecessary controversies.

Clinical trials on the safety and efficacy of melatonin in the field of medicine demonstrate low toxicity with a wide range of doses (Sánchez-Barceló et al., 2010). Doses up to 300 mg/d have been administered over a long period with effective results and no manifestations of adverse effects (Weishaupt et al., 2006), demonstrating its high safety profile. Available in pure pharmacological form, melatonin has a lipid-soluble phase that enables it to be effectively absorbed from all biological membranes (Hardeland, 2005). Additionally, melatonin as an adjuvant of tumor therapy demonstrated tremendous remission of cancer by inducing self-destruction of cells indicating its synergistic potential and also reducing the collateral side effects of chemotherapy drugs (Wang et al., 2012).

The regulatory interaction of melatonin and epigenetics has so far been studied under the conditions of early development. The study of miRNAs interaction with melatonin, though in its infancy, has elicited huge interest in the research field. The signaling pathway of melatonin can be affected by miRNAs, as numerous diseases display alterations in levels and the general composition of miRNAs indicating their importance in the pathology of diseases. Pointing out, the beneficial role of melatonin has been reported in atherosclerosis in elevated levels of miR-29b (Zhu et al., 2014), neoplasm involving miR-374 (Chuffa et al., 2020), memory and cognitive disorders associated with miR-124 (Wang et al., 2013), and so on. However, the direct information available on the action of melatonin to date is missing and does not give a coherent picture as investigations touching on this subject are scattered all over other topics of melatonin. Nevertheless, this field promises to be important as the findings already obtained show miRNAs modulating circadian rhythms (Hardeland, 2014).

## 6 Conclusion

There are but a handful of studies demonstrating the mechanism by which melatonin influences epigenetic processes. Herein, we have highlighted the effects of melatonin on histone modification, DNA methylation, and non-coding RNAs and summarized its significance in alleviating aberrant regulations often leading to diseases. Several other proteins which directly regulate epigenetic modifications causing mutation or functionality loss have resulted in a series of human disorders. This has enabled a precedential focus on the transcription factors and miRNAs in the quest to unlock the mystery behind their association with diseases and melatonin action on them. This, therefore, provides exciting prospects about melatonin as an oscillator of the epigenetic gene in human diseases, and great potential in the development of “epigenetic therapies” as an adjuvant and/or alternative treatment to the currently available therapies.

## References

[B1] Abrams E. W. Andrew D. J. (2002). Prolyl 4-hydroxylase Alpha-Related Proteins in *Drosophila melanogaster*: Tissue-specific Embryonic Expression of the 99F8-9 Cluster. Mech. Dev. 112 (1-2), 165–171. 10.1016/s0925-4773(01)00636-0 11850189

[B2] Acuña-Castroviejo D. Escames G. Venegas C. Díaz-Casado M. E. Lima-Cabello E. López L. C. (2014). Extrapineal Melatonin: Sources, Regulation, and Potential Functions. Cell Mol Life Sci 71 (16), 2997–3025. 10.1007/s00018-014-1579-2 24554058 PMC11113552

[B3] Agbaria S. Haim A. Fares F. Zubidat A. E. (2019). Epigenetic Modification in 4T1 Mouse Breast Cancer Model by Artificial Light at Night and Melatonin - the Role of DNA-Methyltransferase. Chronobiol Int. 36, 629–643. 10.1080/07420528.2019.1574265 30746962

[B4] Aikawa M. Rabkin E. Okada Y. Voglic S. J. Clinton S. K. Brinckerhoff C. E. (1998). Lipid Lowering by Diet Reduces Matrix Metalloproteinase Activity and Increases Collagen Content of Rabbit Atheroma: a Potential Mechanism of Lesion Stabilization. Circulation 97 (24), 2433–2444. 10.1161/01.cir.97.24.2433 9641696

[B5] Akkers R. C. van Heeringen S. J. Jacobi U. G. Janssen-Megens E. M. Françoijs K. J. Stunnenberg H. G. (2009). A Hierarchy of H3K4me3 and H3K27me3 Acquisition in Spatial Gene Regulation in Xenopus Embryos. Dev. Cel 17 (3), 425–434. 10.1016/j.devcel.2009.08.005 PMC2746918 19758566

[B6] Arrighetti N. Cossa G. De Cecco L. Stucchi S. Carenini N. Corna E. (2016). PKC-alpha Modulation by miR-483-3p in Platinum-Resistant Ovarian Carcinoma Cells. Toxicol. Appl. Pharmacol. 310, 9–19. 10.1016/j.taap.2016.08.005 27554045

[B7] Asher G. Gatfield D. Stratmann M. Reinke H. Dibner C. Kreppel F. (2008). SIRT1 Regulates Circadian Clock Gene Expression through PER2 Deacetylation. Cell 134 (2), 317–328. 10.1016/j.cell.2008.06.050 18662546

[B8] Asin-Cayuela J. Gustafsson C. M. (2007). Mitochondrial Transcription and its Regulation in Mammalian Cells. Trends Biochem. Sci. 32 (3), 111–117. 10.1016/j.tibs.2007.01.003 17291767

[B9] Bahna S. G. Niles L. P. (2017). Epigenetic Induction of Melatonin MT1 Receptors by Valproate: Neurotherapeutic Implications. Eur. Neuropsychopharmacol. 27 (8), 828–832. 10.1016/j.euroneuro.2017.06.002 28648552

[B10] Bahna S. G. Niles L. P. (2018). Epigenetic Regulation of Melatonin Receptors in Neuropsychiatric Disorders. Br. J. Pharmacol. 175 (16), 3209–3219. 10.1111/bph.14058 28967098 PMC6057907

[B11] Bansal T. Chatterjee E. Singh J. Ray A. Kundu B. Thankamani V. (2017). Arjunolic Acid, a Peroxisome Proliferator-Activated Receptor α Agonist, Regresses Cardiac Fibrosis by Inhibiting Non-canonical TGF-β Signaling. J. Biol. Chem. 292 (40), 16440–16462. 10.1074/jbc.M117.788299 28821620 PMC5633106

[B12] Baserga M. Kaur R. Hale M. A. Bares A. Yu X. Callaway C. W. (2010). Fetal Growth Restriction Alters Transcription Factor Binding and Epigenetic Mechanisms of Renal 11beta-Hydroxysteroid Dehydrogenase Type 2 in a Sex-specific Manner. Am. J. Physiol. Regul. Integr. Comp. Physiol. 299 (1), R334–R342. 10.1152/ajpregu.00122.2010 20427719 PMC2904157

[B13] Bayraktar G. Kreutz M. R. (2018). Neuronal DNA Methyltransferases: Epigenetic Mediators between Synaptic Activity and Gene Expression? Neuroscientist 24 (2), 171–185. 10.1177/1073858417707457 28513272 PMC5846851

[B14] Bell J. T. Pai A. A. Pickrell J. K. Gaffney D. J. Pique-Regi R. Degner J. F. (2011). DNA Methylation Patterns Associate with Genetic and Gene Expression Variation in HapMap Cell Lines. Genome Biol. 12 (1), R10. 10.1186/gb-2011-12-1-r10 21251332 PMC3091299

[B15] Benke K. K. Benke K. E. (2012). Uncertainty in Health Risks from Artificial Lighting Due to Disruption of Circadian Rhythm and Melatonin Secretion: A Review. Hum. Ecol. Risk Assess. Int. J. 19 (4), 916–929. 10.1080/10807039.2012.702608

[B16] Berson A. Nativio R. Berger S. L. Bonini N. M. (2018). Epigenetic Regulation in Neurodegenerative Diseases. Trends Neurosci. 41 (9), 587–598. 10.1016/j.tins.2018.05.005 29885742 PMC6174532

[B17] Bertelsen D. M. Neergaard J. S. Bager C. L. Nielsen S. H. Secher N. H. Svendsen J. H. (2018). Matrix Metalloproteinase Mediated Type I Collagen Degradation Is an Independent Predictor of Increased Risk of Acute Myocardial Infarction in Postmenopausal Women. Sci. Rep. 8 (1), 5371. 10.1038/s41598-018-23458-4 29599489 PMC5876321

[B18] Bird A. P. (1986). CpG-rich Islands and the Function of DNA Methylation. Nature 321 (6067), 209–213. 10.1038/321209a0 2423876

[B19] Blanch M. Mosquera J. L. Ansoleaga B. Ferrer I. Barrachina M. (2016). Altered Mitochondrial DNA Methylation Pattern in Alzheimer Disease-Related Pathology and in Parkinson Disease. Am. J. Pathol. 186 (2), 385–397. 10.1016/j.ajpath.2015.10.004 26776077

[B20] Bompada P. Atac D. Luan C. Andersson R. Omella J. D. Laakso E. O. (2016). Histone Acetylation of Glucose-Induced Thioredoxin-Interacting Protein Gene Expression in Pancreatic Islets. Int. J. Biochem. Cel Biol 81, 82–91. 10.1016/j.biocel.2016.10.022 27989964

[B21] Bondy S. C. Campbell A. (2018). Mechanisms Underlying Tumor Suppressive Properties of Melatonin. Int. J. Mol. Sci. 19 (8). 10.3390/ijms19082205 PMC6121612 30060531

[B22] Bondy S. C. Sharman E. H. (2007). Melatonin and the Aging Brain. Neurochem. Int. 50 (4), 571–580. 10.1016/j.neuint.2006.12.014 17276551

[B23] Braganza D. M. Bennett M. R. (2001). New Insights into Atherosclerotic Plaque Rupture. Postgrad. Med. J. 77 (904), 94–98. 10.1136/pmj.77.904.94 11161074 PMC1741926

[B24] Brito-Armas J. M. Baekelandt V. Castro-Hernández J. R. González-Hernández T. Rodríguez M. Castro R. (2013). Melatonin Prevents Dopaminergic Cell Loss Induced by Lentiviral Vectors Expressing A30P Mutant Alpha-Synuclein. Histol. Histopathol 28 (8), 999–1006. 10.14670/HH-28.999 23444197

[B25] Butler A. E. Janson J. Bonner-Weir S. Ritzel R. Rizza R. A. Butler P. C. (2003). Beta-cell Deficit and Increased Beta-Cell Apoptosis in Humans with Type 2 Diabetes. Diabetes 52 (1), 102–110. 10.2337/diabetes.52.1.102 12502499

[B26] Cai B. Ma W. Bi C. Yang F. Zhang L. Han Z. (2016). Long Noncoding RNA H19 Mediates Melatonin Inhibition of Premature Senescence of C-Kit(+) Cardiac Progenitor Cells by Promoting miR-675. J. Pineal Res. 61 (1), 82–95. 10.1111/jpi.12331 27062045

[B27] Calin G. A. Dumitru C. D. Shimizu M. Bichi R. Zupo S. Noch E. (2002). Frequent Deletions and Down-Regulation of Micro- RNA Genes miR15 and miR16 at 13q14 in Chronic Lymphocytic Leukemia. Proc. Natl. Acad. Sci. U S A. 99 (24), 15524–15529. 10.1073/pnas.242606799 12434020 PMC137750

[B28] Cardinali D. P. Furio A. M. Brusco L. I. (2010). Clinical Aspects of Melatonin Intervention in Alzheimer's Disease Progression. Curr. Neuropharmacol 8 (3), 218–227. 10.2174/157015910792246209 21358972 PMC3001215

[B29] Cavallari C. Dellepiane S. Fonsato V. Medica D. Marengo M. Migliori M. (2019). Online Hemodiafiltration Inhibits Inflammation-Related Endothelial Dysfunction and Vascular Calcification of Uremic Patients Modulating miR-223 Expression in Plasma Extracellular Vesicles. J. Immunol. 202 (8), 2372–2383. 10.4049/jimmunol.1800747 30833349

[B30] Cerna M. (2019). Epigenetic Regulation in Etiology of Type 1 Diabetes Mellitus. Int. J. Mol. Sci. 21 (1). 10.3390/ijms21010036 PMC6981658 31861649

[B31] Che H. Wang Y. Li H. Li Y. Sahil A. Lv J. (2020). Melatonin Alleviates Cardiac Fibrosis via Inhibiting lncRNA MALAT1/miR-141-Mediated NLRP3 Inflammasome and TGF-β1/Smads Signaling in Diabetic Cardiomyopathy. FASEB J. 34 (4), 5282–5298. 10.1096/fj.201902692R 32067273

[B32] Chen C. C. Chen C. Y. Wang S. H. Yeh C. T. Su S. C. Ueng S. H. (2018). Melatonin Sensitizes Hepatocellular Carcinoma Cells to Chemotherapy through Long Non-coding RNA RAD51-AS1-Mediated Suppression of DNA Repair. Cancers (Basel) 10 (9). 10.3390/cancers10090320 PMC6162454 30201872

[B33] Chen L. Teng H. Fang T. Xiao J. (2016). Agrimonolide from Agrimonia Pilosa Suppresses Inflammatory Responses through Down-Regulation of COX-2/iNOS and Inactivation of NF-Κb in Lipopolysaccharide-Stimulated Macrophages. Phytomedicine 23 (8), 846–855. 10.1016/j.phymed.2016.03.016 27288920

[B34] Chen S. S. Jenkins A. J. Majewski H. (2009). Elevated Plasma Prostaglandins and Acetylated Histone in Monocytes in Type 1 Diabetes Patients. Diabet Med. 26 (2), 182–186. 10.1111/j.1464-5491.2008.02658.x 19236624

[B35] Chen X. Hao A. Li X. Du Z. Li H. Wang H. (2016). Melatonin Inhibits Tumorigenicity of Glioblastoma Stem-like Cells via the AKT-EZH2-STAT3 Signaling axis. J. Pineal Res. 61 (2), 208–217. 10.1111/jpi.12341 27121240

[B36] Chen Y. A. Lu C. H. Ke C. C. Chiu S. J. Chang C. W. Yang B. H. (2021). Evaluation of Class IIa Histone Deacetylases Expression and *In Vivo* Epigenetic Imaging in a Transgenic Mouse Model of Alzheimer's Disease. Int. J. Mol. Sci. 22 (16). 10.3390/ijms22168633 PMC8395513 34445342

[B37] Choi J. Kim K. J. Koh E. J. Lee B. Y. (2018). Gelidium Elegans Extract Ameliorates Type 2 Diabetes via Regulation of MAPK and PI3K/Akt Signaling. Nutrients 10 (1). 10.3390/nu10010051 PMC5793279 29316644

[B38] Chuang C. W. Pan M. R. Hou M. F. Hung W. C. (2013). Cyclooxygenase-2 Up-Regulates CCR7 Expression via AKT-Mediated Phosphorylation and Activation of Sp1 in Breast Cancer Cells. J. Cel Physiol 228 (2), 341–348. 10.1002/jcp.24136 22718198

[B39] Chuang Y. H. Paul K. C. Bronstein J. M. Bordelon Y. Horvath S. Ritz B. (2017). Parkinson's Disease Is Associated with DNA Methylation Levels in Human Blood and Saliva. Genome Med. 9 (1), 76. 10.1186/s13073-017-0466-5 28851441 PMC5576382

[B40] Chuffa L. G. A. Carvalho R. F. Justulin L. A. Cury S. S. Seiva F. R. F. Jardim-Perassi B. V. (2020). A Meta-Analysis of microRNA Networks Regulated by Melatonin in Cancer: Portrait of Potential Candidates for Breast Cancer Treatment. J. Pineal Res. 69 (4), e12693. 10.1111/jpi.12693 32910542

[B41] Clokie S. J. H. Lau P. Kim H. H. Coon S. L. Klein D. C. (2012). MicroRNAs in the Pineal Gland. J. Biol. Chem. 287 (30), 25312–25324. 10.1074/jbc.m112.356733 22908386 PMC3408182

[B42] Costes S. Boss M. Thomas A. P. Matveyenko A. V. (2015). Activation of Melatonin Signaling Promotes β-Cell Survival and Function. Mol. Endocrinol. 29 (5), 682–692. 10.1210/me.2014-1293 25695910 PMC4415205

[B43] Crespo I. San-Miguel B. Fernández A. Ortiz de Urbina J. González-Gallego J. Tuñón M. J. (2015). Melatonin Limits the Expression of Profibrogenic Genes and Ameliorates the Progression of Hepatic Fibrosis in Mice. Transl Res. 165 (2), 346–357. 10.1016/j.trsl.2014.10.003 25445210

[B44] Crowley S. D. Gurley S. B. Herrera M. J. Ruiz P. Griffiths R. Kumar A. P. (2006). Angiotensin II Causes Hypertension and Cardiac Hypertrophy through its Receptors in the Kidney. Proc. Natl. Acad. Sci. U S A. 103 (47), 17985–17990. 10.1073/pnas.0605545103 17090678 PMC1693859

[B45] Daimi H. Lozano-Velasco E. Haj Khelil A. Chibani J. B. Barana A. Amorós I. (2015). Regulation of SCN5A by microRNAs: miR-219 Modulates SCN5A Transcript Expression and the Effects of Flecainide Intoxication in Mice. Heart Rhythm 12 (6), 1333–1342. 10.1016/j.hrthm.2015.02.018 25701775

[B46] Dauchy R. T. Wren-Dail M. A. Dupepe L. M. Hill S. M. Xiang S. Anbalagan M. (2018). Effect of Daytime Blue-Enriched LED Light on the Nighttime Circadian Melatonin Inhibition of Hepatoma 7288CTC Warburg Effect and Progression. Comp. Med. 68 (4), 269–279. 10.30802/AALAS-CM-17-000107 29875029 PMC6103418

[B47] Deng W. G. Tang S. T. Tseng H. P. Wu K. K. (2006). Melatonin Suppresses Macrophage Cyclooxygenase-2 and Inducible Nitric Oxide Synthase Expression by Inhibiting P52 Acetylation and Binding. Blood 108 (2), 518–524. 10.1182/blood-2005-09-3691 16609073 PMC1895491

[B48] Deng W. G. Zhu Y. Wu K. K. (2003). Up-regulation of P300 Binding and P50 Acetylation in Tumor Necrosis Factor-Alpha-Induced Cyclooxygenase-2 Promoter Activation. J. Biol. Chem. 278 (7), 4770–4777. 10.1074/jbc.M209286200 12471036

[B49] Domínguez Rubio A. P. Correa F. Aisemberg J. Dorfman D. Bariani M. V. Rosenstein R. E. (2017). Maternal Administration of Melatonin Exerts Short- and Long-Term Neuroprotective Effects on the Offspring from Lipopolysaccharide-Treated Mice. J. Pineal Res. 63 (4). 10.1111/jpi.12439 28776755

[B50] Doxakis E. (2010). Post-transcriptional Regulation of Alpha-Synuclein Expression by Mir-7 and Mir-153. J. Biol. Chem. 285 (17), 12726–12734. 10.1074/jbc.M109.086827 20106983 PMC2857101

[B51] Drobnik J. Karbownik-Lewińska M. Szczepanowska A. Słotwińska D. Olczak S. Jakubowski L. (2008). Regulatory Influence of Melatonin on Collagen Accumulation in the Infarcted Heart Scar. J. Pineal Res. 45 (3), 285–290. 10.1111/j.1600-079X.2008.00588.x 18384532

[B52] Dupont C. Armant D. R. Brenner C. A. (2009). Epigenetics: Definition, Mechanisms and Clinical Perspective. Semin. Reprod. Med. 27 (5), 351–357. 10.1055/s-0029-1237423 19711245 PMC2791696

[B53] Dwivedi S. K. Mustafi S. B. Mangala L. S. Jiang D. Pradeep S. Rodriguez-Aguayo C. (2016). Therapeutic Evaluation of microRNA-15a and microRNA-16 in Ovarian Cancer. Oncotarget 7 (12), 15093–15104. 10.18632/oncotarget.7618 26918603 PMC4924772

[B54] Edwards J. R. Yarychkivska O. Boulard M. Bestor T. H. (2017). DNA Methylation and DNA Methyltransferases. Epigenetics Chromatin 10, 23. 10.1186/s13072-017-0130-8 28503201 PMC5422929

[B55] Favero G. Moretti E. Bonomini F. Reiter R. J. Rodella L. F. Rezzani R. (2018). Promising Antineoplastic Actions of Melatonin. Front. Pharmacol. 9, 1086. 10.3389/fphar.2018.01086 30386235 PMC6198052

[B56] Fitzsimons H. L. Schwartz S. Given F. M. Scott M. J. (2013). The Histone Deacetylase HDAC4 Regulates Long-Term Memory in Drosophila. PLoS One 8 (12), e83903. 10.1371/journal.pone.0083903 24349558 PMC3857321

[B57] Fuchs O. (2013). Targeting of NF-kappaB Signaling Pathway, Other Signaling Pathways and Epigenetics in Therapy of Multiple Myeloma. Cardiovasc. Hematol. Disord. Drug Targets 13 (1), 16–34. 10.2174/1871529x11313010003 23534949

[B58] Gao Y. Xiao X. Zhang C. Yu W. Guo W. Zhang Z. (2017). Melatonin Synergizes the Chemotherapeutic Effect of 5-fluorouracil in colon Cancer by Suppressing PI3K/AKT and NF-κB/iNOS Signaling Pathways. J. Pineal Res. 62 (2). 10.1111/jpi.12380 27865009

[B59] Garcia-Saenz A. Sánchez de Miguel A. Espinosa A. Valentin A. Aragonés N. Llorca J. (2018). Evaluating the Association between Artificial Light-At-Night Exposure and Breast and Prostate Cancer Risk in Spain (MCC-Spain Study). Environ. Health Perspect. 126 (4), 047011. 10.1289/EHP1837 29687979 PMC6071739

[B60] Giusti-Rodríguez P. Gao J. Gräff J. Rei D. Soda T. Tsai L. H. (2011). Synaptic Deficits Are Rescued in the p25/Cdk5 Model of Neurodegeneration by the Reduction of β-secretase (BACE1). J. Neurosci. 31 (44), 15751–15756. 10.1523/JNEUROSCI.3588-11.2011 22049418 PMC3248793

[B61] Glezeva N. Moran B. Collier P. Moravec C. S. Phelan D. Donnellan E. (2019). Targeted DNA Methylation Profiling of Human Cardiac Tissue Reveals Novel Epigenetic Traits and Gene Deregulation across Different Heart Failure Patient Subtypes. Circ. Heart Fail. 12 (3), e005765. 10.1161/CIRCHEARTFAILURE.118.005765 30798618

[B62] Goh S. Y. Chao Y. X. Dheen S. T. Tan E. K. Tay S. S. (2019). Role of MicroRNAs in Parkinson's Disease. Int. J. Mol. Sci. 20 (22). 10.3390/ijms20225649 PMC6888719 31718095

[B63] Goyal R. Goyal D. Leitzke A. Gheorghe C. P. Longo L. D. (2010). Brain Renin-Angiotensin System: Fetal Epigenetic Programming by Maternal Protein Restriction during Pregnancy. Reprod. Sci. 17 (3), 227–238. 10.1177/1933719109351935 19923380

[B64] Green A. L. Zhan L. Eid A. Zarbl H. Guo G. L. Richardson J. R. (2017). Valproate Increases Dopamine Transporter Expression through Histone Acetylation and Enhanced Promoter Binding of Nurr1. Neuropharmacology 125, 189–196. 10.1016/j.neuropharm.2017.07.020 28743636 PMC5585058

[B65] Grimaldi V. De Pascale M. R. Zullo A. Soricelli A. Infante T. Mancini F. P. (2017). Evidence of Epigenetic Tags in Cardiac Fibrosis. J. Cardiol. 69 (2), 401–408. 10.1016/j.jjcc.2016.10.004 27863907

[B66] Gu J. Lu Z. Ji C. Chen Y. Liu Y. Lei Z. (2017). Melatonin Inhibits Proliferation and Invasion via Repression of miRNA-155 in Glioma Cells. Biomed. Pharmacother. 93, 969–975. 10.1016/j.biopha.2017.07.010 28724215

[B67] Guo X. H. Li Y. H. Zhao Y. S. Zhai Y. Z. Zhang L. C. (2017). Anti-aging E-ffects of M-elatonin on the M-yocardial M-itochondria of R-ats and A-ssociated M-echanisms. Mol. Med. Rep. 15 (1), 403–410. 10.3892/mmr.2016.6002 27959405

[B68] Hammond S. M. Caudy A. A. Hannon G. J. (2001). Post-transcriptional Gene Silencing by Double-Stranded RNA. Nat. Rev. Genet. 2 (2), 110–119. 10.1038/35052556 11253050

[B69] Han C. Zhou J. Liu B. Liang C. Pan X. Zhang Y. (2019). Delivery of miR-675 by Stem Cell-Derived Exosomes Encapsulated in Silk Fibroin Hydrogel Prevents Aging-Induced Vascular Dysfunction in Mouse Hindlimb. Mater. Sci. Eng. C Mater. Biol. Appl. 99, 322–332. 10.1016/j.msec.2019.01.122 30889706

[B70] Hardeland R. (2005). Antioxidative protection by Melatonin: Multiplicity of Mechanisms from Radical Detoxification to Radical Avoidance. Endocrine 27 (2), 119–130. 10.1385/endo:27:2:119 16217125

[B71] Hardeland R. (2012). Melatonin in Aging and Disease -multiple Consequences of Reduced Secretion, Options and Limits of Treatment. Aging Dis. 3 (2), 194–225. 22724080 PMC3377831

[B72] Hardeland R. (2014). Melatonin, Noncoding RNAs, Messenger RNA Stability and Epigenetics-Eevidence, Hints, Gaps and Perspectives. Int. J. Mol. Sci. 15 (10), 18221–18252. 10.3390/ijms151018221 25310649 PMC4227213

[B73] Harrison I. F. Smith A. D. Dexter D. T. (2018). Pathological Histone Acetylation in Parkinson's Disease: Neuroprotection and Inhibition of Microglial Activation through SIRT 2 Inhibition. Neurosci. Lett. 666, 48–57. 10.1016/j.neulet.2017.12.037 29273397 PMC5821898

[B74] He F. Chou C. J. Scheiner M. Poeta E. Yuan Chen N. Gunesch S. (2021). Melatonin- and Ferulic Acid-Based HDAC6 Selective Inhibitors Exhibit Pronounced Immunomodulatory Effects *In Vitro* and Neuroprotective Effects in a Pharmacological Alzheimer's Disease Mouse Model. J. Med. Chem. 64 (7), 3794–3812. 10.1021/acs.jmedchem.0c01940 33769811

[B75] He Z. Cho Y. Y. Ma W. Y. Choi H. S. Bode A. M. Dong Z. (2005). Regulation of Ultraviolet B-Induced Phosphorylation of Histone H3 at Serine 10 by Fyn Kinase. J. Biol. Chem. 280 (4), 2446–2454. 10.1074/jbc.M402053200 15537652

[B76] Henderson-Smith A. Fisch K. M. Hua J. Liu G. Ricciardelli E. Jepsen K. (2019). DNA Methylation Changes Associated with Parkinson's Disease Progression: Outcomes from the First Longitudinal Genome-wide Methylation Analysis in Blood. Epigenetics 14 (4), 365–382. 10.1080/15592294.2019.1588682 30871403 PMC6557551

[B77] Hill S. M. Belancio V. P. Dauchy R. T. Xiang S. Brimer S. Mao L. (2015). Melatonin: an Inhibitor of Breast Cancer. Endocr. Relat. Cancer 22 (3), R183–R204. 10.1530/ERC-15-0030 25876649 PMC4457700

[B78] Hsiao H. M. Wu Y. Y. Tsai B. C. Chen Y. C. Cheng Y. H. (2015). Investigation of Fibrous Cap Stresses on Vulnerable Plaques Leading to Heart Attacks. Technol. Health Care 24 (Suppl. 1), S155–S161. 10.3233/THC-151064 26684564

[B79] Hu G. Gong A. Y. Wang Y. Ma S. Chen X. Chen J. (2016). LincRNA-Cox2 Promotes Late Inflammatory Gene Transcription in Macrophages through Modulating SWI/SNF-Mediated Chromatin Remodeling. J. Immunol. 196 (6), 2799–2808. 10.4049/jimmunol.1502146 26880762 PMC4779692

[B80] Hu W. Ma Z. Jiang S. Fan C. Deng C. Yan X. (2016). Melatonin: the Dawning of a Treatment for Fibrosis? J. Pineal Res. 60 (2), 121–131. 10.1111/jpi.12302 26680689

[B81] Huang X. Lyu J. (2018). Tumor Suppressor Function of miR-483-3p on Breast Cancer via Targeting of the Cyclin E1 Gene. Exp. Ther. Med. 16 (3), 2615–2620. 10.3892/etm.2018.6504 30186493 PMC6122499

[B82] Insull W. Jr. (2009). The Pathology of Atherosclerosis: Plaque Development and Plaque Responses to Medical Treatment. Am. J. Med. 122 (1 Suppl. l), S3–S14. 10.1016/j.amjmed.2008.10.013 19110086

[B83] Jacob-Ferreira A. L. Kondo M. Y. Baral P. K. James M. N. Holt A. Fan X. (2013). Phosphorylation Status of 72 kDa MMP-2 Determines its Structure and Activity in Response to Peroxynitrite. PLoS One 8 (8), e71794. 10.1371/journal.pone.0071794 24013357 PMC3754950

[B84] Jankovic J. (2008). Parkinson's Disease: Clinical Features and Diagnosis. J. Neurol. Neurosurg. Psychiatry 79 (4), 368–376. 10.1136/jnnp.2007.131045 18344392

[B85] Jeong H. Y. Kang W. S. Hong M. H. Jeong H. C. Shin M. G. Jeong M. H. (2015). 5-Azacytidine Modulates Interferon Regulatory Factor 1 in Macrophages to Exert a Cardioprotective Effect. Sci. Rep. 5, 15768. 10.1038/srep15768 26510961 PMC4625165

[B86] Ji Q. Liu X. Han Z. Zhou L. Sui H. Yan L. (2015). Resveratrol Suppresses Epithelial-To-Mesenchymal Transition in Colorectal Cancer through TGF-β1/Smads Signaling Pathway Mediated Snail/E-Cadherin Expression. BMC Cancer 15, 97. 10.1186/s12885-015-1119-y 25884904 PMC4362662

[B87] Jia L. Sun P. Gao H. Shen J. Gao Y. Meng C. (2019). Mangiferin Attenuates Bleomycin-Induced Pulmonary Fibrosis in Mice through Inhibiting TLR4/p65 and TGF-beta1/Smad2/3 Pathway. J. Pharm. Pharmacol. 71, 1017–1028. 10.1111/jphp.13077 30847938

[B88] Jiang J. Liang S. Zhang J. Du Z. Xu Q. Duan J. (2020). Melatonin Ameliorates PM2.5 -induced Cardiac Perivascular Fibrosis through Regulating Mitochondrial Redox Homeostasis. J. Pineal Res., e12686. 10.1111/jpi.12686 32730639 PMC7757260

[B89] Ju H. Q. Li H. Tian T. Lu Y. X. Bai L. Chen L. Z. (2016). Melatonin Overcomes Gemcitabine Resistance in Pancreatic Ductal Adenocarcinoma by Abrogating Nuclear Factor-Κb Activation. J. Pineal Res. 60 (1), 27–38. 10.1111/jpi.12285 26445000

[B90] Kamat A. M. Cookson M. Witjes J. A. Stenzl A. Grossman H. B. (2016). The Impact of Blue Light Cystoscopy with Hexaminolevulinate (HAL) on Progression of Bladder Cancer - A New Analysis. Bladder Cancer 2 (2), 273–278. 10.3233/BLC-160048 27376146 PMC4927917

[B91] Kellogg A. P. Cheng H. T. Pop-Busui R. (2008). Cyclooxygenase-2 Pathway as a Potential Therapeutic Target in Diabetic Peripheral Neuropathy. Curr. Drug Targets 9 (1), 68–76. 10.2174/138945008783431691 18220714

[B92] Kim J. M. Heo H. S. Ha Y. M. Ye B. H. Lee E. K. Choi Y. J. (2012). Mechanism of Ang II Involvement in Activation of NF-Κb through Phosphorylation of P65 during Aging. Age (Dordr) 34 (1), 11–25. 10.1007/s11357-011-9207-7 21318332 PMC3260361

[B93] Kim T. Song S. Park Y. Kang S. Seo H. (2019). HDAC Inhibition by Valproic Acid Induces Neuroprotection and Improvement of PD-like Behaviors in LRRK2 R1441G Transgenic Mice. Exp. Neurobiol. 28 (4), 504–515. 10.5607/en.2019.28.4.504 31495079 PMC6751862

[B94] Kong J. Zhang Y. Liu S. Li H. Liu S. Wang J. (2017). Melatonin Attenuates Angiotensin II-Induced Abdominal Aortic Aneurysm through the Down-Regulation of Matrix Metalloproteinases. Oncotarget 8 (9), 14283–14293. 10.18632/oncotarget.15093 28179581 PMC5362405

[B95] Kong P. Christia P. Frangogiannis N. G. (2014). The Pathogenesis of Cardiac Fibrosis. Cel Mol Life Sci 71 (4), 549–574. 10.1007/s00018-013-1349-6 PMC3769482 23649149

[B96] Krell A. Wolter M. Stojcheva N. Hertler C. Liesenberg F. Zapatka M. (2018). MiR-16-5p Is Frequently Down-Regulated in Astrocytic Gliomas and Modulates Glioma Cell Proliferation, Apoptosis and Response to Cytotoxic Therapy. Neuropathol. Appl. Neurobiol. 45, 441–458. 10.1111/nan.12532 30548945

[B97] Labbé C. Lorenzo-Betancor O. Ross O. A. (2016). Epigenetic Regulation in Parkinson's Disease. Acta Neuropathol. 132 (4), 515–530. 10.1007/s00401-016-1590-9 27358065 PMC5026906

[B98] Lahiri D. K. Ge Y. W. Sharman E. H. Bondy S. C. (2004). Age-related Changes in Serum Melatonin in Mice: Higher Levels of Combined Melatonin and 6-hydroxymelatonin Sulfate in the Cerebral Cortex Than Serum, Heart, Liver and Kidney Tissues. J. Pineal Res. 36 (4), 217–223. 10.1111/j.1600-079X.2004.00120.x 15066045

[B99] Lee B. Shim I. Lee H. Hahm D. H. (2018). Melatonin Ameliorates Cognitive Memory by Regulation of cAMP-Response Element-Binding Protein Expression and the Anti-inflammatory Response in a Rat Model of post-traumatic Stress Disorder. BMC Neurosci. 19 (1), 38. 10.1186/s12868-018-0439-7 29973144 PMC6032787

[B100] Lee I. C. Ko J. W. Park S. H. Shin N. R. Shin I. S. Moon C. (2018). Copper Nanoparticles Induce Early Fibrotic Changes in the Liver via TGF-β/Smad Signaling and Cause Immunosuppressive Effects in Rats. Nanotoxicology 12 (6), 637–651. 10.1080/17435390.2018.1472313 29848140

[B101] Li B. Xie Z. Li B. (2016). miR-152 Functions as a Tumor Suppressor in Colorectal Cancer by Targeting PIK3R3. Tumour Biol. 37 (8), 10075–10084. 10.1007/s13277-016-4888-2 26820128

[B102] Li H. Li J. Jiang X. Liu S. Liu Y. Chen W. (2019). Melatonin Enhances Atherosclerotic Plaque Stability by Inducing Prolyl-4-Hydroxylase α1 Expression. J. Hypertens. 37 (5), 964–971. 10.1097/HJH.0000000000001979 30335670

[B103] Li K. Hu F. Xiong W. Wei Q. Liu F. F. (2019). Network-based Transcriptomic Analysis Reveals Novel Melatonin-Sensitive Genes in Cardiovascular System. Endocrine 64 (2), 414–419. 10.1007/s12020-019-01925-w 30989468

[B104] Li S. Chen H. Ren J. Geng Q. Song J. Lee C. (2014). MicroRNA-223 Inhibits Tissue Factor Expression in Vascular Endothelial Cells. Atherosclerosis 237 (2), 514–520. 10.1016/j.atherosclerosis.2014.09.033 25463083

[B105] Li T. Ni L. Zhao Z. Liu X. Lai Z. Di X. (2018). Melatonin Attenuates Smoking-Induced Hyperglycemia via Preserving Insulin Secretion and Hepatic Glycogen Synthesis in Rats. J. Pineal Res. 64 (4), e12475. 10.1111/jpi.12475 29437243 PMC5947659

[B106] Li W. Q. Tan S. L. Li X. H. Sun T. L. Li D. Du J. (2019). Calcitonin Gene-Related Peptide Inhibits the Cardiac Fibroblasts Senescence in Cardiac Fibrosis via Up-Regulating Klotho Expression. Eur. J. Pharmacol. 843, 96–103. 10.1016/j.ejphar.2018.10.023 30352200

[B107] Li X. Chen X. Zhou W. Ji S. Li X. Li G. (2017). Effect of Melatonin on Neuronal Differentiation Requires CBP/p300-mediated Acetylation of Histone H3 Lysine 14. Neuroscience 364, 45–59. 10.1016/j.neuroscience.2017.07.064 28782640

[B108] Li X. W. Cao L. Wang F. Yang Q. G. Tong J. J. Li X. Y. (2016). Maternal Inflammation Linearly Exacerbates Offspring Age-Related Changes of Spatial Learning and Memory, and Neurobiology until Senectitude. Behav. Brain Res. 306, 178–196. 10.1016/j.bbr.2016.03.011 26992827

[B109] Li Y. Li S. Zhou Y. Meng X. Zhang J. J. Xu D. P. (2017). Melatonin for the Prevention and Treatment of Cancer. Oncotarget 8 (24), 39896–39921. 10.18632/oncotarget.16379 28415828 PMC5503661

[B110] Liang H. Xu Y. Zhang Q. Yang Y. Mou Y. Gao Y. (2019). MiR-483-3p Regulates Oxaliplatin Resistance by Targeting FAM171B in Human Colorectal Cancer Cells. Artif. Cell Nanomed Biotechnol 47 (1), 725–736. 10.1080/21691401.2019.1569530 30861353

[B111] Liao W. W. Zhang C. Liu F. R. Wang W. J. (2018). Effects of miR-155 on Proliferation and Apoptosis by Regulating FoxO3a/BIM in Liver Cancer Cell Line HCCLM3. Eur. Rev. Med. Pharmacol. Sci. 22 (5), 1277–1285. 10.26355/eurrev_201803_14468 29565484

[B112] Libby P. Aikawa M. (2003). Mechanisms of Plaque Stabilization with Statins. Am. J. Cardiol. 91 (4A), 4B–8B. 10.1016/s0002-9149(02)03267-8 12615292

[B113] Lin G. J. Huang S. H. Chen Y. W. Hueng D. Y. Chien M. W. Chia W. T. (2009). Melatonin Prolongs Islet Graft Survival in Diabetic NOD Mice. J. Pineal Res. 47 (3), 284–292. 10.1111/j.1600-079X.2009.00712.x 19708865

[B114] Lind M. I. Spagopoulou F. (2018). Evolutionary Consequences of Epigenetic Inheritance. Heredity (Edinb) 121 (3), 205–209. 10.1038/s41437-018-0113-y 29976958 PMC6082883

[B115] Ling J. Kang Y. Zhao R. Xia Q. Lee D. F. Chang Z. (2012). KrasG12D-induced IKK2/β/NF-Κb Activation by IL-1α and P62 Feedforward Loops Is Required for Development of Pancreatic Ductal Adenocarcinoma. Cancer Cell 21 (1), 105–120. 10.1016/j.ccr.2011.12.006 22264792 PMC3360958

[B116] Ling J. Kang Y. a. Zhao R. Xia Q. Lee D.-F. Chang Z. (2012). KrasG12D-Induced IKK2/β/NF-Κb Activation by IL-1α and P62 Feedforward Loops Is Required for Development of Pancreatic Ductal Adenocarcinoma. Cancer Cell 21 (1), 105–120. 10.1016/j.ccr.2011.12.006 22264792 PMC3360958

[B117] Litwin M. Michałkiewicz J. Trojanek J. Niemirska A. Wierzbicka A. Szalecki M. (2013). Altered Genes Profile of Renin-Angiotensin System, Immune System, and Adipokines Receptors in Leukocytes of Children with Primary Hypertension. Hypertension 61 (2), 431–436. 10.1161/HYPERTENSIONAHA.111.00181 23266543

[B118] Liu T. Zhang L. Joo D. Sun S. C. (2017). NF-κB Signaling in Inflammation. Signal. Transduct Target. Ther. 2. 10.1038/sigtrans.2017.23 PMC5661633 29158945

[B119] Liu X. Cheng Y. Yang J. Krall T. J. Huo Y. Zhang C. (2010). An Essential Role of PDCD4 in Vascular Smooth Muscle Cell Apoptosis and Proliferation: Implications for Vascular Disease. Am. J. Physiol. Cel Physiol 298 (6), C1481–C1488. 10.1152/ajpcell.00413.2009 PMC2889634 20357187

[B120] Liu Y. Li L. N. Guo S. Zhao X. Y. Liu Y. Z. Liang C. (2018). Melatonin Improves Cardiac Function in a Mouse Model of Heart Failure with Preserved Ejection Fraction. Redox Biol. 18, 211–221. 10.1016/j.redox.2018.07.007 30031269 PMC6076208

[B121] Long F. Dong C. Jiang K. Xu Y. Chi X. Sun D. (2017). Melatonin Enhances the Anti-tumor Effect of Sorafenib via AKT/p27-mediated Cell Cycle Arrest in Hepatocarcinoma Cell Lines. RSC Adv. 7 (34), 21342–21351. 10.1039/c7ra02113e

[B122] Lupini L. Pepe F. Ferracin M. Braconi C. Callegari E. Pagotto S. (2016). Over-expression of the miR-483-3p Overcomes the miR-145/TP53 Pro-apoptotic Loop in Hepatocellular Carcinoma. Oncotarget 7 (21), 31361–31371. 10.18632/oncotarget.8913 27120784 PMC5058762

[B123] Lustri A. M. Di Matteo S. Fraveto A. Costantini D. Cantafora A. Napoletano C. (2017). TGF-β Signaling Is an Effective Target to Impair Survival and Induce Apoptosis of Human Cholangiocarcinoma Cells: A Study on Human Primary Cell Cultures. PLoS One 12 (9), e0183932. 10.1371/journal.pone.0183932 28873435 PMC5584931

[B124] Lyu G. Guan Y. Zhang C. Zong L. Sun L. Huang X. (2018). TGF-β Signaling Alters H4K20me3 Status via miR-29 and Contributes to Cellular Senescence and Cardiac Aging. Nat. Commun. 9 (1), 2560. 10.1038/s41467-018-04994-z 29967491 PMC6028646

[B125] Ma J. Hong L. Xu G. Hao J. Wang R. Guo H. (2016). miR-483-3p Plays an Oncogenic Role in Esophageal Squamous Cell Carcinoma by Targeting Tumor Suppressor EI24. Cell Biol Int 40 (4), 448–455. 10.1002/cbin.10585 26801660

[B126] Majd S. Power J. H. T. Koblar S. A. Grantham H. J. M. (2017). The Impact of Tau Hyperphosphorylation at Ser262 on Memory and Learning after Global Brain Ischaemia in a Rat Model of Reversible Cardiac Arrest. IBRO Rep. 2, 1–13. 10.1016/j.ibror.2016.12.002 30135928 PMC6084925

[B127] Makeeva N. Myers J. W. Welsh N. (2006). Role of MKK3 and P38 MAPK in Cytokine-Induced Death of Insulin-Producing Cells. Biochem. J. 393 (Pt 1), 129–139. 10.1042/BJ20050814 16097952 PMC1383671

[B128] Marques J. H. M. Mota A. L. Oliveira J. G. Lacerda J. Z. Stefani J. P. Ferreira L. C. (2018). Melatonin Restrains Angiogenic Factors in Triple-Negative Breast Cancer by Targeting miR-152-3p: *In Vivo* and *In Vitro* Studies. Life Sci. 208, 131–138. 10.1016/j.lfs.2018.07.012 29990486

[B129] Martino F. Carlomosti F. Avitabile D. Persico L. Picozza M. Barillà F. (2015). Circulating miR-33a and miR-33b Are Up-Regulated in Familial Hypercholesterolaemia in Paediatric Age. Clin. Sci. (Lond) 129 (11), 963–972. 10.1042/CS20150235 26229086

[B130] Medeiros C. A. Carvalhedo de Bruin P. F. Lopes L. A. Magalhães M. C. de Lourdes Seabra M. de Bruin V. M. (2007). Effect of Exogenous Melatonin on Sleep and Motor Dysfunction in Parkinson's Disease. A Randomized, Double Blind, Placebo-Controlled Study. J. Neurol. 254 (4), 459–464. 10.1007/s00415-006-0390-x 17404779

[B131] Medina M. Garrido J. J. Wandosell F. G. (2011). Modulation of GSK-3 as a Therapeutic Strategy on Tau Pathologies. Front. Mol. Neurosci. 4, 24. 10.3389/fnmol.2011.00024 22007157 PMC3186940

[B132] Monti N. Cavallaro R. A. Stoccoro A. Nicolia V. Scarpa S. Kovacs G. G. (2020). CpG and Non-CpG Presenilin1 Methylation Pattern in Course of Neurodevelopment and Neurodegeneration Is Associated with Gene Expression in Human and Murine Brain. Epigenetics 15 (8), 781–799. 10.1080/15592294.2020.1722917 32019393 PMC7518704

[B133] Moriya J. (2019). Critical Roles of Inflammation in Atherosclerosis. J. Cardiol. 73 (1), 22–27. 10.1016/j.jjcc.2018.05.010 29907363

[B134] Ohashi N. Ishigaki S. Isobe S. (2019). The Pivotal Role of Melatonin in Ameliorating Chronic Kidney Disease by Suppression of the Renin-Angiotensin System in the Kidney. Hypertens. Res. 42 (6), 761–768. 10.1038/s41440-018-0186-2 30610209

[B135] Ohashi N. Ishigaki S. Isobe S. (2019). The Pivotal Role of Melatonin in Ameliorating Chronic Kidney Disease by Suppression of the Renin-Angiotensin System in the Kidney. Hypertens. Res. 10.1038/s41440-018-0186-2 30610209

[B136] Olcese J. M. Cao C. Mori T. Mamcarz M. B. Maxwell A. Runfeldt M. J. (2009). Protection against Cognitive Deficits and Markers of Neurodegeneration by Long-Term Oral Administration of Melatonin in a Transgenic Model of Alzheimer Disease. J. Pineal Res. 47 (1), 82–96. 10.1111/j.1600-079X.2009.00692.x 19538338

[B137] Ono K. Mochizuki H. Ikeda T. Nihira T. Takasaki J. Teplow D. B. (2012). Effect of Melatonin on α-synuclein Self-Assembly and Cytotoxicity. Neurobiol. Aging 33 (9), 2172–2185. 10.1016/j.neurobiolaging.2011.10.015 22118903

[B138] Ortiz G. G. Pacheco-Moisés F. P. Gómez-Rodríguez V. M. González-Renovato E. D. Torres-Sánchez E. D. Ramírez-Anguiano A. C. (2013). Fish Oil, Melatonin and Vitamin E Attenuates Midbrain Cyclooxygenase-2 Activity and Oxidative Stress after the Administration of 1-Methyl-4-Phenyl-1,2,3,6- Tetrahydropyridine. Metab. Brain Dis. 28 (4), 705–709. 10.1007/s11011-013-9416-0 23703110

[B139] Ozsoy O. Yildirim F. B. Ogut E. Kaya Y. Tanriover G. Parlak H. (2015). Melatonin Is Protective against 6-Hydroxydopamine-Induced Oxidative Stress in a Hemiparkinsonian Rat Model. Free Radic. Res. 49 (8), 1004–1014. 10.3109/10715762.2015.1027198 25791066

[B140] Pan Y. Niles L. P. (2015). Epigenetic Mechanisms of Melatonin Action in Human SH-Sy5y Neuroblastoma Cells. Mol. Cel Endocrinol 402, 57–63. 10.1016/j.mce.2015.01.003 25578604

[B141] Park G. Tan J. Garcia G. Kang Y. Salvesen G. Zhang Z. (2016). Regulation of Histone Acetylation by Autophagy in Parkinson Disease. J. Biol. Chem. 291 (7), 3531–3540. 10.1074/jbc.M115.675488 26699403 PMC4751393

[B142] Park J. H. Shim H. M. Na A. Y. Bae K. C. Bae J. H. Im S. S. (2014). Melatonin Prevents Pancreatic β-cell Loss Due to Glucotoxicity: the Relationship between Oxidative Stress and Endoplasmic Reticulum Stress. J. Pineal Res. 56 (2), 143–153. 10.1111/jpi.12106 24168371

[B143] Park M. H. Hong J. T. (2016). Roles of NF-Κb in Cancer and Inflammatory Diseases and Their Therapeutic Approaches. Cells 5 (2). 10.3390/cells5020015 PMC4931664 27043634

[B144] Paul R. Phukan B. C. Justin Thenmozhi A. Manivasagam T. Bhattacharya P. Borah A. (2018). Melatonin Protects against Behavioral Deficits, Dopamine Loss and Oxidative Stress in Homocysteine Model of Parkinson's Disease. Life Sci. 192, 238–245. 10.1016/j.lfs.2017.11.016 29138117

[B145] Paz S. Vilasco M. Werden S. J. Arguello M. Joseph-Pillai D. Zhao T. (2011). A Functional C-Terminal TRAF3-Binding Site in MAVS Participates in Positive and Negative Regulation of the IFN Antiviral Response. Cell Res 21 (6), 895–910. 10.1038/cr.2011.2 21200404 PMC3203699

[B146] Pei F. Wang X. Yue R. Chen C. Huang J. Huang J. (2015). Differential Expression and DNA Methylation of Angiotensin Type 1A Receptors in Vascular Tissues during Genetic Hypertension Development. Mol. Cel Biochem 402 (1-2), 1–8. 10.1007/s11010-014-2295-9 25596947

[B147] Pestana R. M. Domingueti C. P. Duarte R. C. Fóscolo R. B. Reis J. S. Rodrigues A. M. (2016). Cytokines Profile and its Correlation with Endothelial Damage and Oxidative Stress in Patients with Type 1 Diabetes Mellitus and Nephropathy. Immunol. Res. 64 (4), 951–960. 10.1007/s12026-016-8806-x 27307060

[B148] Pihlstrøm L. Berge V. Rengmark A. Toft M. (2015). Parkinson's Disease Correlates with Promoter Methylation in the α-synuclein Gene. Mov Disord. 30 (4), 577–580. 10.1002/mds.26073 25545759

[B149] Pore N. Liu S. Shu H. K. Li B. Haas-Kogan D. Stokoe D. (2004). Sp1 Is Involved in Akt-Mediated Induction of VEGF Expression through an HIF-1-independent Mechanism. Mol. Biol. Cel 15 (11), 4841–4853. 10.1091/mbc.e04-05-0374 PMC524732 15342781

[B150] Prokopenko I. Langenberg C. Florez J. C. Saxena R. Soranzo N. Thorleifsson G. (2009). Variants in MTNR1B Influence Fasting Glucose Levels. Nat. Genet. 41 (1), 77–81. 10.1038/ng.290 19060907 PMC2682768

[B151] Qin W. Lu W. Li H. Yuan X. Li B. Zhang Q. (2012). Melatonin Inhibits IL1β-induced MMP9 Expression and Activity in Human Umbilical Vein Endothelial Cells by Suppressing NF-Κb Activation. J. Endocrinol. 214 (2), 145–153. 10.1530/JOE-12-0147 22619232

[B152] Qin Z. Fisher G. J. Voorhees J. J. Quan T. (2018). Actin Cytoskeleton Assembly Regulates Collagen Production via TGF-β Type II Receptor in Human Skin Fibroblasts. J. Cel Mol Med 22 (9), 4085–4096. 10.1111/jcmm.13685 PMC6111811 29888864

[B153] Renani P. G. Taheri F. Rostami D. Farahani N. Abdolkarimi H. Abdollahi E. (2019). Involvement of Aberrant Regulation of Epigenetic Mechanisms in the Pathogenesis of Parkinson's Disease and Epigenetic-Based Therapies. J. Cel Physiol 234 (11), 19307–19319. 10.1002/jcp.28622 30968426

[B154] Rinnerthaler G. Hackl H. Gampenrieder S. P. Hamacher F. Hufnagl C. Hauser-Kronberger C. (2016). miR-16-5p Is a Stably-Expressed Housekeeping MicroRNA in Breast Cancer Tissues from Primary Tumors and from Metastatic Sites. Int. J. Mol. Sci. 17 (2). 10.3390/ijms17020156 PMC4783890 26821018

[B155] Roelofs H. M. Te Morsche R. H. van Heumen B. W. Nagengast F. M. Peters W. H. (2014). Over-expression of COX-2 mRNA in Colorectal Cancer. BMC Gastroenterol. 14, 1. 10.1186/1471-230X-14-1 24383454 PMC3880419

[B156] Rossetto D. Avvakumov N. Côté J. (2012). Histone Phosphorylation: a Chromatin Modification Involved in Diverse Nuclear Events. Epigenetics 7 (10), 1098–1108. 10.4161/epi.21975 22948226 PMC3469451

[B157] Rudra D. S. Pal U. Maiti N. C. Reiter R. J. Swarnakar S. (2013). Melatonin Inhibits Matrix Metalloproteinase-9 Activity by Binding to its Active Site. J. Pineal Res. 54 (4), 398–405. 10.1111/jpi.12034 23330737

[B158] Rui J. Deng S. Lebastchi J. Clark P. L. Usmani-Brown S. Herold K. C. (2016). Methylation of Insulin DNA in Response to Proinflammatory Cytokines during the Progression of Autoimmune Diabetes in NOD Mice. Diabetologia 59 (5), 1021–1029. 10.1007/s00125-016-3897-4 26910463 PMC4826795

[B159] Ruiz L. Gurlo T. Ravier M. A. Wojtusciszyn A. Mathieu J. Brown M. R. (2018). Proteasomal Degradation of the Histone Acetyl Transferase P300 Contributes to Beta-Cell Injury in a Diabetes Environment. Cell Death Dis 9 (6), 600. 10.1038/s41419-018-0603-0 29789539 PMC5964068

[B160] Rusek M. Pluta R. Ułamek-Kozioł M. Czuczwar S. J. (2019). Ketogenic Diet in Alzheimer's Disease. Int. J. Mol. Sci. 20 (16). 10.3390/ijms20163892 PMC6720297 31405021

[B161] Russart K. L. G. Nelson R. J. (2018). Light at Night as an Environmental Endocrine Disruptor. Physiol. Behav. 190, 82–89. 10.1016/j.physbeh.2017.08.029 28870443 PMC5839924

[B162] Saldeen J. Lee J. C. Welsh N. (2001). Role of P38 Mitogen-Activated Protein Kinase (P38 MAPK) in Cytokine-Induced Rat Islet Cell Apoptosis. Biochem. Pharmacol. 61 (12), 1561–1569. 10.1016/s0006-2952(01)00605-0 11377386

[B163] Sánchez D. I. González-Fernández B. Crespo I. San-Miguel B. Álvarez M. González-Gallego J. (2018). Melatonin Modulates Dysregulated Circadian Clocks in Mice with Diethylnitrosamine-Induced Hepatocellular Carcinoma. J. Pineal Res. 65 (3), e12506. 10.1111/jpi.12506 29770483

[B164] Sánchez-Barceló E. J. Mediavilla M. D. Tan D. X. Reiter R. J. (2010). Clinical Uses of Melatonin: Evaluation of Human Trials. Curr. Med. Chem. 17 (19), 2070–2095. 10.2174/092986710791233689 20423309

[B165] Sang S. Zhang Z. Qin S. Li C. Dong Y. (2017). MicroRNA-16-5p Inhibits Osteoclastogenesis in Giant Cell Tumor of Bone. Biomed. Res. Int. 2017, 3173547. 10.1155/2017/3173547 28589137 PMC5447262

[B166] Sariahmetoglu M. Crawford B. D. Leon H. Sawicka J. Li L. Ballermann B. J. (2007). Regulation of Matrix Metalloproteinase-2 (MMP-2) Activity by Phosphorylation. FASEB J. 21 (10), 2486–2495. 10.1096/fj.06-7938com 17435175

[B167] Sarshad A. A. Juan A. H. Muler A. I. C. Anastasakis D. G. Wang X. Genzor P. (2018). Argonaute-miRNA Complexes Silence Target mRNAs in the Nucleus of Mammalian Stem Cells. Mol. Cel 71 (6), 1040–e8. 10.1016/j.molcel.2018.07.020 PMC6690358 30146314

[B168] Sawicka A. Seiser C. (2012). Histone H3 Phosphorylation - a Versatile Chromatin Modification for Different Occasions. Biochimie 94 (11), 2193–2201. 10.1016/j.biochi.2012.04.018 22564826 PMC3480636

[B169] Schmitt I. Kaut O. Khazneh H. deBoni L. Ahmad A. Berg D. (2015). L-Dopa Increases α-synuclein DNA Methylation in Parkinson's Disease Patients *In Vivo* and *In Vitro* . Mov Disord. 30 (13), 1794–1801. 10.1002/mds.26319 26173746

[B170] Sharma S. Taliyan R. (2015). Targeting Histone Deacetylases: a Novel Approach in Parkinson's Disease. Parkinsons Dis. 2015, 303294. 10.1155/2015/303294 25694842 PMC4324954

[B171] Shi C. Zeng J. Li Z. Chen Q. Hang W. Xia L. (2018). Melatonin Mitigates Kainic Acid-Induced Neuronal Tau Hyperphosphorylation and Memory Deficits through Alleviating ER Stress. Front. Mol. Neurosci. 11, 5. 10.3389/fnmol.2018.00005 29416502 PMC5787934

[B172] Shi D. Xiao X. Wang J. Liu L. Chen W. Fu L. (2012). Melatonin Suppresses Proinflammatory Mediators in Lipopolysaccharide-Stimulated CRL1999 Cells via Targeting MAPK, NF-Κb, c/EBPβ, and P300 Signaling. J. Pineal Res. 53 (2), 154–165. 10.1111/j.1600-079X.2012.00982.x 22348531

[B173] Shrestha S. Zhu J. Wang Q. Du X. Liu F. Jiang J. (2017). Melatonin Potentiates the Antitumor Effect of Curcumin by Inhibiting IKKβ/NF-Κb/cox-2 Signaling Pathway. Int. J. Oncol. 51 (4), 1249–1260. 10.3892/ijo.2017.4097 28849163 PMC5592853

[B174] Shukla M. Chinchalongporn V. Govitrapong P. Reiter R. J. (2019). The Role of Melatonin in Targeting Cell Signaling Pathways in Neurodegeneration. Ann. N. Y Acad. Sci. 1443 (1), 75–96. 10.1111/nyas.14005 30756405

[B175] Shukla M. Htoo H. H. Wintachai P. Hernandez J. F. Dubois C. Postina R. (2015). Melatonin Stimulates the Nonamyloidogenic Processing of βAPP through the Positive Transcriptional Regulation of ADAM10 and ADAM17. J. Pineal Res. 58 (2), 151–165. 10.1111/jpi.12200 25491598

[B176] Shukla S. Tekwani B. L. (2020). Histone Deacetylases Inhibitors in Neurodegenerative Diseases, Neuroprotection and Neuronal Differentiation. Front. Pharmacol. 11, 537. 10.3389/fphar.2020.00537 32390854 PMC7194116

[B177] Singh P. Konar A. Kumar A. Srivas S. Thakur M. K. (2015). Hippocampal Chromatin-Modifying Enzymes Are Pivotal for Scopolamine-Induced Synaptic Plasticity Gene Expression Changes and Memory Impairment. J. Neurochem. 134 (4), 642–651. 10.1111/jnc.13171 25982413

[B178] Su L. Y. Li H. Lv L. Feng Y. M. Li G. D. Luo R. (2015). Melatonin Attenuates MPTP-Induced Neurotoxicity via Preventing CDK5-Mediated Autophagy and SNCA/α-synuclein Aggregation. Autophagy 11 (10), 1745–1759. 10.1080/15548627.2015.1082020 26292069 PMC4824603

[B179] Su W. Huo Q. Wu H. Wang L. Ding X. Liang L. (2021). The Function of LncRNA-H19 in Cardiac Hypertrophy. Cell Biosci 11 (1), 153. 10.1186/s13578-021-00668-4 34344446 PMC8336097

[B180] Sun J. Tian X. Zhang J. Huang Y. Lin X. Chen L. (2017). Regulation of Human Glioma Cell Apoptosis and Invasion by miR-152-3p through Targeting DNMT1 and Regulating NF2 : MiR-152-3p Regulate Glioma Cell Apoptosis and Invasion. J. Exp. Clin. Cancer Res. 36 (1), 100. 10.1186/s13046-017-0567-4 28764788 PMC5539621

[B181] Sun X. Ran D. Zhao X. Huang Y. Long S. Liang F. (2016). Melatonin Attenuates hLRRK2-Induced Sleep Disturbances and Synaptic Dysfunction in a Drosophila Model of Parkinson's Disease. Mol. Med. Rep. 13 (5), 3936–3944. 10.3892/mmr.2016.4991 26985725

[B182] Sun Y. Jin L. Sui Y. X. Han L. L. Liu J. H. (2017). Circadian Gene CLOCK Affects Drug-Resistant Gene Expression and Cell Proliferation in Ovarian Cancer SKOV3/DDP Cell Lines through Autophagy. Cancer Biother. Radiopharm. 32 (4), 139–146. 10.1089/cbr.2016.2153 28514207

[B183] Sung H. Ferlay J. Siegel R. L. Laversanne M. Soerjomataram I. Jemal A. (2021). Global Cancer Statistics 2020: GLOBOCAN Estimates of Incidence and Mortality Worldwide for 36 Cancers in 185 Countries. CA Cancer J. Clin. 71 (3), 209–249. 10.3322/caac.21660 33538338

[B184] Taheri M. Eghtedarian R. Dinger M. E. Ghafouri-Fard S. (2020). Emerging Roles of Non-coding RNAs in the Pathogenesis of Type 1 Diabetes Mellitus. Biomed. Pharmacother. 129, 110509. 10.1016/j.biopha.2020.110509 32768981

[B185] Tai N. Wong F. S. Wen L. (2016). The Role of the Innate Immune System in Destruction of Pancreatic Beta Cells in NOD Mice and Humans with Type I Diabetes. J. Autoimmun. 71, 26–34. 10.1016/j.jaut.2016.03.006 27021275 PMC4903935

[B186] Tain Y. L. Chan J. Y. H. Lee C. T. Hsu C. N. (2018). Maternal Melatonin Therapy Attenuates Methyl-Donor Diet-Induced Programmed Hypertension in Male Adult Rat Offspring. Nutrients 10 (10). 10.3390/nu10101407 PMC6213858 30279341

[B187] Tain Y. L. Lee C. T. Chan J. Y. Hsu C. N. (2016). Maternal Melatonin or N-Acetylcysteine Therapy Regulates hydrogen Sulfide-Generating Pathway And renal transcriptome to Prevent Prenatal NG-Nitro-L-arginine-methyl Ester (L-NAME)-induced Fetal Programming of Hypertension in Adult Male Offspring. Am. J. Obstet. Gynecol. 215 (5), 636–e72. 10.1016/j.ajog.2016.07.036 27457113

[B188] Tain Y. L. Lin Y. J. Chan J. Y. H. Lee C. T. Hsu C. N. (2017). Maternal Melatonin or Agomelatine Therapy Prevents Programmed Hypertension in Male Offspring of Mother Exposed to Continuous Light. Biol. Reprod. 97 (4), 636–643. 10.1093/biolre/iox115 29025027

[B189] Tang Y. L. Jiang J. H. Wang S. Liu Z. Tang X. Q. Peng J. (2015). TLR4/NF-κB Signaling Contributes to Chronic Unpredictable Mild Stress-Induced Atherosclerosis in ApoE-/- Mice. PLoS One 10 (4), e0123685. 10.1371/journal.pone.0123685 25860573 PMC4393302

[B190] Taniguchi K. Karin M. (2018). NF-κB, Inflammation, Immunity and Cancer: Coming of Age. Nat. Rev. Immunol. 18 (5), 309–324. 10.1038/nri.2017.142 29379212

[B191] Tao H. Yang J. J. Shi K. H. Li J. (2016). Epigenetic Factors MeCP2 and HDAC6 Control α-tubulin Acetylation in Cardiac Fibroblast Proliferation and Fibrosis. Inflamm. Res. 65 (5), 415–426. 10.1007/s00011-016-0925-2 26975406

[B192] Vallabhapurapu S. D. Noothi S. K. Pullum D. A. Lawrie C. H. Pallapati R. Potluri V. (2015). Transcriptional Repression by the HDAC4-RelB-P52 Complex Regulates Multiple Myeloma Survival and Growth. Nat. Commun. 6, 8428. 10.1038/ncomms9428 26455434

[B193] Wang C. Sun Y. Wu H. Yu S. Zhang L. Meng Y. (2015). Elevated miR-483-3p Expression Is an Early Event and Indicates Poor Prognosis in Pancreatic Ductal Adenocarcinoma. Tumour Biol. 36 (12), 9447–9456. 10.1007/s13277-015-3690-x 26124009

[B194] Wang D. B. Kinoshita C. Kinoshita Y. Sopher B. L. Uo T. Lee R. J. (2019). Neuronal Susceptibility to Beta-Amyloid Toxicity and Ischemic Injury Involves Histone Deacetylase-2 Regulation of Endophilin-B1. Brain Pathol. 29 (2), 164–175. 10.1111/bpa.12647 30028551 PMC6339848

[B195] Wang F. Demura M. Cheng Y. Zhu A. Karashima S. Yoneda T. (2014). Dynamic CCAAT/enhancer Binding Protein-Associated Changes of DNA Methylation in the Angiotensinogen Gene. Hypertension 63 (2), 281–288. 10.1161/HYPERTENSIONAHA.113.02303 24191285

[B196] Wang F. Mao A. Tang J. Zhang Q. Yan J. Wang Y. (2019). microRNA-16-5p Enhances Radiosensitivity through Modulating Cyclin D1/E1-pRb-E2f1 Pathway in Prostate Cancer Cells. J. Cel Physiol 234 (Suppl. ment_1), 13182–13190. 10.1002/jcp.27989 PMC13483218 30536619

[B197] Wang H. Lou D. Wang Z. (2018). Crosstalk of Genetic Variants, Allele-specific DNA Methylation, and Environmental Factors for Complex Disease Risk. Front. Genet. 9, 695. 10.3389/fgene.2018.00695 30687383 PMC6334214

[B198] Wang H. W. Shi L. Xu Y. P. Qin X. Y. Wang Q. Z. (2016). Oxymatrine Inhibits Renal Fibrosis of Obstructive Nephropathy by Downregulating the TGF-Β1-Smad3 Pathway. Ren. Fail. 38 (6), 945–951. 10.3109/0886022X.2016.1164185 27050799

[B199] Wang R. Zhou S. Wu P. Li M. Ding X. Sun L. (2018). Identifying Involvement of H19-miR-675-3p-Igf1r and H19-miR-200a-PDCD4 in Treating Pulmonary Hypertension with Melatonin. Mol. Ther. Nucleic Acids 13, 44–54. 10.1016/j.omtn.2018.08.015 30240970 PMC6146608

[B200] Wang X. Tang S. Le S. Y. Lu R. Rader J. S. Meyers C. (2008). Aberrant Expression of Oncogenic and Tumor-Suppressive microRNAs in Cervical Cancer Is Required for Cancer Cell Growth. PLoS One 3 (7), e2557. 10.1371/journal.pone.0002557 18596939 PMC2438475

[B201] Wang X. Wang Z. H. Wu Y. Y. Tang H. Tan L. Wang X. (2013). Melatonin Attenuates Scopolamine-Induced Memory/synaptic Disorder by Rescuing EPACs/miR-124/Egr1 Pathway. Mol. Neurobiol. 47 (1), 373–381. 10.1007/s12035-012-8355-9 23054680

[B202] Wang X. X. Xie F. Jia C. C. Yan N. Zeng Y. L. Wu J. D. (2021). Synthesis and Biological Evaluation of Selective Histone Deacetylase 6 Inhibitors as Multifunctional Agents against Alzheimer's Disease. Eur. J. Med. Chem. 225, 113821. 10.1016/j.ejmech.2021.113821 34517222

[B203] Wang Y. M. Jin B. Z. Ai F. Duan C. H. Lu Y. Z. Dong T. F. (2012). The Efficacy and Safety of Melatonin in Concurrent Chemotherapy or Radiotherapy for Solid Tumors: a Meta-Analysis of Randomized Controlled Trials. Cancer Chemother. Pharmacol. 69 (5), 1213–1220. 10.1007/s00280-012-1828-8 22271210

[B204] Wang Y. R. Hong R. T. Xie Y. Y. Xu J. M. (2018). Melatonin Ameliorates Liver Fibrosis Induced by Carbon Tetrachloride in Rats via Inhibiting TGF-β1/Smad Signaling Pathway. Curr. Med. Sci. 38 (2), 236–244. 10.1007/s11596-018-1871-8 30074181

[B205] Weishaupt J. H. Bartels C. Pölking E. Dietrich J. Rohde G. Poeggeler B. (2006). Reduced Oxidative Damage in ALS by High-Dose Enteral Melatonin Treatment. J. Pineal Res. 41 (4), 313–323. 10.1111/j.1600-079X.2006.00377.x 17014688

[B206] Wijesekara N. Krishnamurthy M. Bhattacharjee A. Suhail A. Sweeney G. Wheeler M. B. (2010). Adiponectin-induced ERK and Akt Phosphorylation Protects against Pancreatic Beta Cell Apoptosis and Increases Insulin Gene Expression and Secretion. J. Biol. Chem. 285 (44), 33623–33631. 10.1074/jbc.M109.085084 20709750 PMC2962460

[B207] Wu T. H. Kuo H. C. Lin I. C. Chien S. J. Huang L. T. Tain Y. L. (2014). Melatonin Prevents Neonatal Dexamethasone Induced Programmed Hypertension: Histone Deacetylase Inhibition. J. Steroid Biochem. Mol. Biol. 144, 253–259. 10.1016/j.jsbmb.2014.07.008 25090636

[B208] Wu W. L. Wang W. Y. Yao W. Q. Li G. D. (2015). Suppressive Effects of microRNA-16 on the Proliferation, Invasion and Metastasis of Hepatocellular Carcinoma Cells. Int. J. Mol. Med. 36 (6), 1713–1719. 10.3892/ijmm.2015.2379 26499886

[B209] Wu X. Chen P. S. Dallas S. Wilson B. Block M. L. Wang C. C. (2008). Histone Deacetylase Inhibitors Up-Regulate Astrocyte GDNF and BDNF Gene Transcription and Protect Dopaminergic Neurons. Int. J. Neuropsychopharmacol. 11 (8), 1123–1134. 10.1017/S1461145708009024 18611290 PMC2579941

[B210] Wu Y. Si F. Luo L. Jing F. Jiang K. Zhou J. (2018). The Effect of Melatonin on Cardio Fibrosis in Juvenile Rats with Pressure Overload and Deregulation of HDACs. Korean J. Physiol. Pharmacol. 22 (6), 607–616. 10.4196/kjpp.2018.22.6.607 30402021 PMC6205940

[B211] Xu J. Gao H. Zhang L. Rong S. Yang W. Ma C. (2019). Melatonin Alleviates Cognition Impairment by Antagonizing Brain Insulin Resistance in Aged Rats Fed a High-Fat Diet. J. Pineal Res. 67, e12584. 10.1111/jpi.12584 31050371

[B212] Xu X. Wang G. Ai L. Shi J. Zhang J. Chen Y. X. (2018). Melatonin Suppresses TLR9-Triggered Proinflammatory Cytokine Production in Macrophages by Inhibiting ERK1/2 and AKT Activation. Sci. Rep. 8 (1), 15579. 10.1038/s41598-018-34011-8 30349079 PMC6197220

[B213] Yan W. W. Chen G. H. Wang F. Tong J. J. Tao F. (2015). Long-term Acarbose Administration Alleviating the Impairment of Spatial Learning and Memory in the SAMP8 Mice Was Associated with Alleviated Reduction of Insulin System and Acetylated H4K8. Brain Res. 1603, 22–31. 10.1016/j.brainres.2015.01.042 25645154

[B214] Yang M. Tao J. Wu H. Guan S. Liu L. Zhang L. (2019). Aanat Knockdown and Melatonin Supplementation in Embryo Development: Involvement of Mitochondrial Function and DNA Methylation. Antioxid. Redox Signal. 30 (18), 2050–2065. 10.1089/ars.2018.7555 30343588

[B215] Yeung H. M. Hung M. W. Lau C. F. Fung M. L. (2015). Cardioprotective Effects of Melatonin against Myocardial Injuries Induced by Chronic Intermittent Hypoxia in Rats. J. Pineal Res. 58 (1), 12–25. 10.1111/jpi.12190 25369321

[B216] Yi C. Zhang Y. Yu Z. Xiao Y. Wang J. Qiu H. (2014). Melatonin Enhances the Anti-tumor Effect of Fisetin by Inhibiting COX-2/iNOS and NF-κB/p300 Signaling Pathways. PLoS One 9 (7), e99943. 10.1371/journal.pone.0099943 25000190 PMC4085069

[B217] You D. Qiao Q. Ono K. Wei M. Tan W. Wang C. (2022). miR-223-3p Inhibits the Progression of Atherosclerosis via Down-Regulating the Activation of MEK1/ERK1/2 in Macrophages. Aging (Albany NY) 14 (4), 1865–1878. 10.18632/aging.203908 35202001 PMC8908932

[B218] Yu D. Du Z. Pian L. Li T. Wen X. Li W. (2017). Mitochondrial DNA Hypomethylation Is a Biomarker Associated with Induced Senescence in Human Fetal Heart Mesenchymal Stem Cells. Stem Cell Int 2017, 1764549. 10.1155/2017/1764549 PMC5397648 28484495

[B219] Yu L. M. Xu Y. (2015). Epigenetic Regulation in Cardiac Fibrosis. World J. Cardiol. 7 (11), 784–791. 10.4330/wjc.v7.i11.784 26635926 PMC4660473

[B220] Zablocki D. Sadoshima J. (2013). Angiotensin II and Oxidative Stress in the Failing Heart. Antioxid. Redox Signal. 19 (10), 1095–1109. 10.1089/ars.2012.4588 22429089 PMC3771547

[B221] Zhang H. Yang K. Ren T. Huang Y. Tang X. Guo W. (2018). miR-16-5p Inhibits Chordoma Cell Proliferation, Invasion and Metastasis by Targeting Smad3. Cel Death Dis 9 (6), 680. 10.1038/s41419-018-0738-z PMC5992191 29880900

[B222] Zhang J. Song Y. Zhang C. Zhi X. Fu H. Ma Y. (2015). Circulating MiR-16-5p and MiR-19b-3p as Two Novel Potential Biomarkers to Indicate Progression of Gastric Cancer. Theranostics 5 (7), 733–745. 10.7150/thno.10305 25897338 PMC4402497

[B223] Zhang L. Wang W. Li X. He S. Yao J. Wang X. (2016). MicroRNA-155 Promotes Tumor Growth of Human Hepatocellular Carcinoma by Targeting ARID2. Int. J. Oncol. 48 (6), 2425–2434. 10.3892/ijo.2016.3465 27035278

[B224] Zhang X. Liu L. Deng X. Li D. Cai H. Ma Y. (2019). MicroRNA 483-3p Targets Pard3 to Potentiate TGF-Β1-Induced Cell Migration, Invasion, and Epithelial-Mesenchymal Transition in Anaplastic Thyroid Cancer Cells. Oncogene 38 (5), 699–715. 10.1038/s41388-018-0447-1 30171257 PMC6756112

[B225] Zhang Y. Liu X. Bai X. Lin Y. Li Z. Fu J. (2018). Melatonin Prevents Endothelial Cell Pyroptosis via Regulation of Long Noncoding RNA MEG3/miR-223/NLRP3 axis. J. Pineal Res. 64 (2). 10.1111/jpi.12449 29024030

[B226] Zhao Y. Gong X. Chen L. Li L. Liang Y. Chen S. (2014). Site-specific Methylation of Placental HSD11B2 Gene Promoter Is Related to Intrauterine Growth Restriction. Eur. J. Hum. Genet. 22 (6), 734–740. 10.1038/ejhg.2013.226 24129435 PMC4023205

[B227] Zhao Y. Jaber V. Lukiw W. J. (2017). Secretory Products of the Human GI Tract Microbiome and Their Potential Impact on Alzheimer's Disease (AD): Detection of Lipopolysaccharide (LPS) in AD Hippocampus. Front Cel Infect Microbiol 7, 318. 10.3389/fcimb.2017.00318 PMC5504724 28744452

[B228] Zhong S. Goto H. Inagaki M. Dong Z. (2003). Phosphorylation at Serine 28 and Acetylation at Lysine 9 of Histone H3 Induced by Trichostatin A. Oncogene 22 (34), 5291–5297. 10.1038/sj.onc.1206507 12917630

[B229] Zhu C. Huang Q. Zhu H. (2018). Melatonin Inhibits the Proliferation of Gastric Cancer Cells through Regulating the miR-16-5p-Smad3 Pathway. DNA Cel Biol 37 (3), 244–252. 10.1089/dna.2017.4040 29359963

[B230] Zhu H. Q. li Q. Dong L. Y. Zhou Q. Wang H. Wang Y. (2014). MicroRNA-29b Promotes High-Fat Diet-Stimulated Endothelial Permeability and Apoptosis in apoE Knock-Out Mice by Down-Regulating MT1 Expression. Int. J. Cardiol. 176 (3), 764–770. 10.1016/j.ijcard.2014.07.095 25131924

[B231] Zhuang Y. Li T. Xiao H. Wu J. Su S. Dong X. (2021). LncRNA-H19 Drives Cardiomyocyte Senescence by Targeting miR-19a/socs1/p53 Axis. Front. Pharmacol. 12, 631835. 10.3389/fphar.2021.631835 33664669 PMC7921730

[B232] Zibolka J. Mühlbauer E. Peschke E. (2015). Melatonin Influences Somatostatin Secretion from Human Pancreatic δ-cells via MT1 and MT2 Receptors. J. Pineal Res. 58 (2), 198–209. 10.1111/jpi.12206 25585597

[B233] Zou X. Wang J. Chen C. Tan X. Huang Y. Jose P. A. (2020). Secreted Monocyte miR-27a, via Mesenteric Arterial Mas Receptor-eNOS Pathway, Causes Hypertension. Am. J. Hypertens. 33 (1), 31–42. 10.1093/ajh/hpz112 31328772 PMC8205426

[B234] Zou Z. W. Liu T. Li Y. Chen P. Peng X. Ma C. (2018). Melatonin Suppresses Thyroid Cancer Growth and Overcomes Radioresistance via Inhibition of P65 Phosphorylation and Induction of ROS. Redox Biol. 16, 226–236. 10.1016/j.redox.2018.02.025 29525603 PMC5854931

